# Ellagic Acid Promotes Repair of Acetic Acid-Induced Oral Ulcers in Rats: Associations with Redox-Inflammatory Responses and STAT3/EGFR Signaling

**DOI:** 10.3390/antiox15101275

**Published:** 2026-10-02

**Authors:** Jianfa Wu, Ying Cui, Huanang Chen, Shaofeng Shuai, Zhimin Zhou, Sijing Su, Yusen Hou, Ce Tang, Yi Zhang

**Affiliations:** 1 State Key Laboratory of Southwestern Chinese Medicine Resources, School of Pharmacy, Chengdu University of Traditional Chinese Medicine, Chengdu 611137, China; wujianfa0626@stu.cdutcm.edu.cn (J.W.); ccuiy1110@stu.cdutcm.edu.cn (Y.C.); tangce@cdutcm.edu.cn (C.T.); 2 Lab for Innovation & Effective Uses of Chinese Drug Germplasm Resources, School of Pharmacy, Chengdu University of Traditional Chinese Medicine, Chengdu 611137, China; 3 School of Modern Chinese Medicine Industry, Chengdu University of Traditional Chinese Medicine, Chengdu 611137, China; 4 School of Ethnic Medicine, Chengdu University of Traditional Chinese Medicine, Chengdu 611137, China

**Keywords:** ellagic acid, oral ulcer, oral mucosal repair, STAT3, EGFR, oxidative stress

## Abstract

Oxidative stress and inflammation are central barriers to oral mucosal ulcer repair, yet redox-oriented interventions for recurrent aphthous ulcer (RAU)-relevant injury remain insufficiently defined. Ellagic acid (EA) is a naturally occurring polyphenol with antioxidant and anti-inflammatory activities, but its role in oral mucosal repair and the associated signaling events require further characterization. Here, EA was selected from serum-exposed constituents of Yumai Kouyan Heji (YMKY) and evaluated using computational target prioritization, a rat model of acetic acid-induced oral ulcers, and H_2_O_2_-injured human oral keratinocytes. Network and molecular analyses identified STAT3 and EGFR as putative targets for further investigation. In vivo, EA accelerated ulcer closure, improved epithelial integrity and collagen organization, improved serum antioxidant indices, reduced serum MDA and inflammatory cytokine levels, and attenuated apoptosis. EA also reduced injury-associated phosphorylation of STAT3 and EGFR in ulcer tissues. In vitro, EA improved cell viability and migration-assay outcomes in H_2_O_2_-injured human oral keratinocytes, reduced DCF fluorescence and inflammatory responses, preserved mitochondrial membrane potential, reduced apoptosis, and regulated STAT3/EGFR phosphorylation together with Bax/Bcl-2 expression. These findings support the repair-promoting activity of EA in experimental oral mucosal injury, accompanied by improved redox-inflammatory indices and altered STAT3/EGFR signaling. The causal involvement of these pathways and the applicability of the findings to human RAU remain to be established.

## 1. Introduction

Recurrent aphthous ulcers (RAUs), also known as recurrent aphthous stomatitis, are common inflammatory lesions of the oral mucosa characterized by painful, recurrent ulceration on non-keratinized mucosal surfaces [1]. Although individual lesions usually heal within 1–2 weeks, repeated episodes can markedly impair eating, speaking, oral hygiene, and quality of life [2,3]. The pathogenesis of RAU is multifactorial and involves genetic susceptibility, local trauma, psychological stress, nutritional deficiency, hormonal changes, and systemic immune-related disorders [4]. These triggers converge on epithelial injury, immune activation, and delayed mucosal repair. In this context, oxidative stress is increasingly recognized as a key contributor to ulcer progression because excessive reactive oxygen species (ROS) can amplify inflammatory signaling, damage epithelial cells, and disturb the transition from tissue injury to repair.

Redox-inflammatory imbalance provides a biologically plausible framework for understanding impaired oral mucosal healing. Excessive ROS production promotes lipid peroxidation, mitochondrial dysfunction, and apoptosis, while also enhancing the release of inflammatory mediators such as TNF-α, IL-6, and IL-1β [5,6]. These inflammatory mediators further aggravate epithelial damage and delay re-epithelialization, creating a self-reinforcing cycle of oxidative stress and mucosal inflammation. Therefore, restoration of redox homeostasis may represent a repair-oriented strategy for RAU-relevant oral mucosal injury, rather than a purely anti-inflammatory or symptomatic intervention.

Signal transducer and activator of transcription 3 (STAT3) and epidermal growth factor receptor (EGFR) are closely involved in the balance between inflammatory injury and epithelial repair. STAT3 is activated by multiple pro-inflammatory cytokines and can amplify local inflammatory responses when persistently phosphorylated [7,8]. EGFR, in contrast, is essential for epithelial proliferation, migration, and re-epithelialization during wound repair [9]. Notably, STAT3 and EGFR are functionally interconnected, and their coordinated regulation is important for the transition from inflammation to tissue regeneration [10,11]. Redox imbalance may further influence this axis by enhancing cytokine-driven STAT3 activation, impairing epithelial survival, and disrupting EGFR-dependent repair signaling [12,13]. Thus, the STAT3/EGFR pathway may serve as a signaling interface linking oxidative-inflammatory injury to oral mucosal repair.

Ellagic acid (EA) is a naturally occurring polyphenolic antioxidant found in medicinal and edible plants, including ellagitannin-rich botanical sources such as *Phyllanthus emblica* [14]. EA and its metabolites have been associated with antioxidant, anti-inflammatory, anti-apoptotic, and epithelial-protective activities [15,16]. These properties correspond well to the oxidative stress, inflammatory activation, apoptosis, and delayed re-epithelialization observed in oral mucosal ulcer injury. EA has also shown protective effects in experimental oral mucositis, supporting its relevance to ulcerative oral mucosal damage [17]. In the present study, serum pharmacochemistry of Yumai Kouyan Heji (YMKY) was used as a source-tracing strategy to identify bioavailable prototype constituents, and EA was selected for further mechanistic evaluation. We aimed to determine whether EA promotes repair in RAU-relevant oral mucosal injury by restoring redox-inflammatory homeostasis and modulating injury-associated STAT3/EGFR signaling.

## 2. Materials and Methods

### 2.1. Sample Preparation

EA (purity ≥ 98%) was used for all in vivo and in vitro interventions (Chengdu Lemeitian Pharmaceutical Technology Co., Ltd., Chengdu, China). To trace its chemical origin in the formulation, YMKY extract was analyzed by UPLC-Q Exactive-Orbitrap HRMS for prototype constituents detectable in rat serum. YMKY was supplied by Yunnan Shengke Pharmaceutical Co., Ltd. (Kunming, China). The formulation comprises the fruits of *Phyllanthus emblica*, the tuberous roots of *Ophiopogon japonicus*, the tuberous roots of *Rehmannia glutinosa*, the roots of *Paeonia lactiflora*, and the roots of *Glycyrrhiza uralensis*. YMKY was used for chemical profiling and serum pharmacochemical analysis to identify constituents detectable in the circulation following administration, providing a basis for selecting EA for subsequent investigation. Briefly, the mixed herbs were decocted twice with water, and the combined decoctions were filtered, concentrated, lyophilized, and stored at −80 °C before analysis.

Chromatographic separation was achieved on an Accucore™ C_18_ column (3 mm × 100 mm, 2.6 μm) at 30 °C. The injection volume was 3 μL, and the flow rate was 0.4 mL/min. The mobile phases were 0.1% formic acid in water (A) and acetonitrile (B). The elution gradient was set as follows: 0–5 min, 0% B; 5–10 min, 0–7% B; 10–15 min, 15% B; 15–20 min, 15–20% B; 20–35 min, 20–30% B; 35–40 min, 30–40% B; 40–45 min, 40–80% B; and 45–55 min, 80–95% B.

The mass spectrometer was operated with electrospray ionization (ESI) in both positive and negative ion modes. The spray voltage was set at +3.5 kV/−3.0 kV. The auxiliary gas heater temperature was 350 °C, with a sheath gas flow rate of 40 arb, an auxiliary gas flow rate of 15 arb, and an ion transfer tube temperature of 300 °C. Data were collected in full-scan MS with data-dependent MS/MS acquisition. The resolutions were 35,000 for full MS and 17,500 for MS/MS. The scan range was 100–1500 *m*/*z*, and stepped collision energies were set to 20, 40, and 60 eV. Raw data were processed using Compound Discoverer 3.2 and Xcalibur Qual Browser 4.6 (Thermo Fisher Scientific, Waltham, MA, USA).

### 2.2. Serum Pharmacochemistry

All animal experimental protocols were reviewed and approved by the Experimental Animal Welfare Ethics Committee of Chengdu University of Traditional Chinese Medicine (Approval No. 2025769). All animal procedures were conducted in accordance with the approved protocol and relevant institutional guidelines and regulations. For serum pharmacochemical analysis, rats were randomly assigned to a YMKY-treated group or a control group (*n* = 6 per group). Rats in the YMKY-treated group received YMKY extract by intragastric administration at 3 g/kg/day for five consecutive days, whereas rats in the control group received an equal volume of distilled water. After the final administration, blood samples were collected at 15, 30, 45, 60, 80, and 120 min. The collected blood was allowed to clot at room temperature for 1 h and then centrifuged at 12,000× *g* for 15 min at 4 °C. Serum was collected from the supernatant and pooled across the six sampling time points to obtain representative medicated and control serum samples. The pooled serum was mixed with nine volumes of a prechilled methanol–acetonitrile mixture (1:1, *v*/*v*), vortexed for 5 min, and incubated in an ice bath for 10 min. Following centrifugation at 12,000× *g* for 15 min at 4 °C, the supernatant was collected and evaporated to dryness under nitrogen. The residue was reconstituted in 500 μL of 70% methanol and centrifuged before the supernatant was injected for UPLC-Q Exactive-Orbitrap HRMS analysis. This experiment was performed to identify prototype constituents absorbed into the circulation and to provide a chemical basis for selecting EA for subsequent validation.

Medicated and control serum samples were processed identically and analyzed using the chromatographic and mass spectrometric conditions described above.

### 2.3. Prediction of Drug-Likeness

The drug-likeness properties of the serum-absorbed constituents were evaluated using the SwissADME online tool (http://www.swissadme.ch/) (accessed on 12 December 2025) [18]. In addition, the absorption, distribution, metabolism, excretion, and toxicity (ADMET) characteristics of these constituents were predicted using the preADMET web-based platform (https://preadmet.webservice.bmdrc.org/) (accessed on 12 December 2025) [19].

### 2.4. Network Pharmacology Analysis

Potential targets of the serum-absorbed prototype constituents were predicted using SwissTargetPrediction (http://www.swisstargetprediction.ch/) (accessed on 12 December 2025) [20], and duplicate entries were removed. RAU-associated genes were collected from GeneCards (https://www.genecards.org/) [21] and OMIM (https://omim.org/) (accessed on 12 December 2025) [22]. The overlapping targets between the absorbed-constituent target set and the RAU-associated gene set were identified and imported into the STRING database (https://string-db.org/) [23] to generate a protein–protein interaction (PPI) network. The network was then visualized and topologically analyzed in Cytoscape 3.8.0 [24]. Hub targets were screened according to network centrality. GO and KEGG enrichment analyses were then performed to characterize the biological processes and signaling pathways associated with the shared targets, with emphasis on inflammation, oxidative stress, epithelial repair, and receptor-mediated signaling.

### 2.5. Molecular Docking and Molecular Dynamics (MD) Simulations

Based on the serum pharmacochemistry and network pharmacology results, the absorbed prototype constituents were docked against the hub targets STAT3 and EGFR. EA was then selected for further MD simulation because it showed the most favorable docking profile against both targets.

#### 2.5.1. Ligand and Receptor Preparation

The 2D and 3D structures of the candidate serum-absorbed constituents were retrieved from *PubChem* in SDF format. Geometry optimization was performed using the MM2 force field, and the optimized ligands were saved in PDB format. Crystal structures of the target proteins were retrieved from the Protein Data Bank (PDB; https://www.rcsb.org/) [25]. Proteins were prepared by removing crystallographic water molecules and co-crystallized ligands, adding polar hydrogens, and assigning Gasteiger charges in AutoDockTools 1.5.7 (The Scripps Research Institute, La Jolla, CA, USA). The ligand torsions were set to be rotatable, and both receptors and ligands were converted to PDBQT format.

#### 2.5.2. Molecular Docking

Molecular docking was performed using AutoDock Vina 1.2.5 (The Scripps Research Institute, La Jolla, CA, USA) [26]. The grid boxes for STAT3 and EGFR were defined using active-site residues identified from the interaction profile of the reference compound for STAT3 and EGFR was defined using active-site residues identified from the interaction profile of the reference compound. The active-site residues used to define the grid boxes were as follows: STAT3 (ASP570, ASP566, ARG335, PRO333, ASP334, and HIS332) and EGFR (LEU679, ARG681, LEU754, ALA743, MET742, and VAL745). The grid box centers were set at (x, y, z) = (27.629365, −12.048981, 53.427971) for STAT3 and (7.643186, 34.072408, 19.784683) for EGFR. Docking conformations were ranked based on predicted binding affinity (kcal/mol). Visual analysis of the highest-scoring conformations with reasonable binding orientations was performed using PyMOL 3.0.3 and R 4.5.2, focusing on hydrogen bonds, hydrophobic interactions, π-π stacking, and salt bridges.

#### 2.5.3. MD Simulation

To further interrogate the intermolecular contacts and dynamic stability of the protein-ligand complex, MD simulations were carried out starting from the complex conformation provided. All simulations were performed in GROMACS 2022 [27]. The protein was described using the Amber99sb-ildn force field, while ligand parameters and topology were generated using the general Amber force field. The complex was solvated with explicit water using the extended simple point charge model in a simulation box sufficiently large to fully enclose the entire system, and counter-ions were introduced to achieve charge neutrality and automatically balance the ionic environment. Long-range electrostatic interactions were treated using the particle-mesh Ewald method. Prior to equilibration, the system underwent energy minimization using the steepest descent algorithm with a maximum of 500,000 steps; both Coulomb and van der Waals interactions employed a cutoff of 1.0 nm. The minimized system was then equilibrated sequentially under the NVT ensemble for 100 ps followed by the NPT ensemble for 100 ps. During the production phase, temperature was maintained at 298.15 K using the V-rescale thermostat and pressure was controlled at 1 bar under periodic boundary conditions. Finally, a 100 ns production simulation was performed for each complex for subsequent trajectory analyses.

### 2.6. Density Functional Theory (DFT) Analysis

All quantum-chemical calculations were performed within the framework of DFT using the Gaussian 16 program package. The geometries of the investigated molecules were fully optimized at the Becke, three-parameter, Lee-Yang-Parr hybrid functional level with Grimme’s D3 dispersion correction with Becke-Johnson damping [28], in combination with the 6-31G* basis set. Based on the optimized structures, Mulliken atomic charges and the density of states (DOS) were evaluated using the Multiwfn program [29]. The isosurface plots of the highest occupied molecular orbital (HOMO) and the lowest unoccupied molecular orbital (LUMO), as well as the molecular electrostatic potential (MEP) mapped on the molecular surface, were generated and visualized using GaussView 6.0.16 (Semichem Inc., Shawnee Mission, KS, USA).

### 2.7. Animal Grouping and EA Treatment

All animal procedures were approved by the Experimental Animal Welfare Ethics Committee of Chengdu University of Traditional Chinese Medicine (Ethical No. 2025769). Male Sprague-Dawley rats weighing 250–280 g were obtained from SPF (Beijing, China) Biotechnology Co., Ltd. Rats were housed at 22 ± 2 °C and 50–60% humidity under a 12 h light/dark cycle, with free access to food and water. After 7 days of acclimatization, animals were randomly assigned to six groups (*n* = 6 per group): control, model, compound chlorhexidine-dexamethasone patch (CCDP), EA low-dose (EA-LD, 10 mg/kg/day), EA medium-dose (EA-MD, 25 mg/kg/day), and EA high-dose (EA-HD, 50 mg/kg/day) groups. All groups received the same pelleted laboratory rodent diet supplied by Beijing HFK Bioscience Co., Ltd. (Beijing, China).

#### 2.7.1. RAU Model Establishment

A chemically induced oral ulcer model was established according to a previously reported method with minor modifications [30]. Briefly, animals were anesthetized with tribromoethanol (DW3120). A circular filter paper disc (5 mm in diameter) soaked in 60% acetic acid was applied to the buccal mucosa for 30 s. The treated area was then rinsed thoroughly with sterile saline. Ulcer formation was evaluated 24 h after induction.

This chemically induced oral ulcer model was used to reproduce key mucosal injury and repair features relevant to RAU, including epithelial disruption, inflammatory activation, oxidative stress, and delayed re-epithelialization. However, it does not model the recurrent nature or full immune etiology of human RAU.

#### 2.7.2. Drug Administration

EA was administered by intragastric gavage once daily for 7 consecutive days, beginning 24 h after ulcer induction. EA was freshly prepared as a suspension in 0.5% carboxymethylcellulose sodium. Control and model rats received the same vehicle at an equivalent gavage volume. The CCDP group received compound chlorhexidine-dexamethasone patches. Outcome assessors were blinded to group allocation whenever possible. After treatment, animals were euthanized under deep anesthesia. Blood and ulcer tissue samples were collected for biochemical, histological, and immunofluorescence analyses.

### 2.8. Cell Culture, Grouping, and Treatment

#### 2.8.1. Cell Culture

Human oral keratinocytes (HOK cells) were obtained from Otwo Biotech Inc. (Guangzhou, China) and maintained in high-glucose Dulbecco’s modified Eagle’s medium supplemented with 10% fetal bovine serum and 1% penicillin-streptomycin. Cells were cultured at 37 °C in a humidified incubator containing 5% CO_2_. The medium was changed every 2–3 d, and cells in the logarithmic growth phase were used for subsequent experiments.

#### 2.8.2. Cell Model Establishment

To establish an in vitro oxidative injury model, HOK cells were exposed to H_2_O_2_ for 24 h. Before formal experiments, different concentrations of H_2_O_2_ were tested, and cell viability was assessed using a 3-(4,5-dimethylthiazol-2-yl)-2,5-diphenyltetrazolium bromide assay kit (Beyotime Biotech Inc., Shanghai, China) to determine the modeling condition that induced stable oxidative injury. Briefly, after treatment, the assay reagent was added to each well and incubated according to the manufacturer’s instructions. The formazan product was dissolved, and absorbance was measured using a microplate reader. Cell viability was calculated relative to the control group.

#### 2.8.3. Dose Optimization and Drug Administration

EA was dissolved in 1 M NaOH to prepare a stock solution and diluted with culture medium before use. The corresponding NaOH vehicle was included to evaluate the potential effect of the solvent on HOK cells. Dexamethasone solution (DXMS; Beijing Labgic Technology Co., Ltd., Beijing, China) was used as the positive control at a final concentration of 1 μM. Before formal intervention experiments, the effects of different concentrations of EA and the corresponding vehicle on HOK cell viability were evaluated. EA intervention concentrations were further screened in H_2_O_2_-injured HOK cells. Based on these preliminary results, HOK cells were assigned to six groups: control, model, low-dose EA (EA-LD), medium-dose EA (EA-MD), high-dose EA (EA-HD), and DXMS. The Model group was treated with H_2_O_2_ alone. The EA-LD, EA-MD, EA-HD, and DXMS groups received the corresponding interventions after model establishment. The Control group was cultured under normal conditions and received the corresponding vehicle. After treatment, cells, cell lysates, and culture supernatants were collected for subsequent analyses. For each cellular assay, 3 independent experiments were performed using separately prepared and treated HOK cell cultures, with 3–6 technical replicate wells per condition within each experiment. The numbers of independent experiments and technical replicates are specified for each assay in the corresponding figure legends.

### 2.9. Ulcer Area Calculation

The ulcer sites were photographed at fixed time points (days 1, 3, 5, and 7) using a digital camera under standardized lighting conditions and at a fixed distance, with a millimeter scale placed adjacent to each lesion for calibration. Ulcer areas (mm^2^) were quantified using ImageJ 1.54p software (National Institutes of Health, Bethesda, MD, USA) by manually outlining the ulcer boundary.

### 2.10. Determination of Antioxidant Enzymes and Malondialdehyde (MDA) Content

Blood samples were collected at the indicated time point, allowed to clot at room temperature, and centrifuged to obtain serum. The serum samples were stored at −80 °C until analysis. For cell samples, HOK cells were collected after the indicated treatments, washed twice with ice-cold phosphate-buffered saline (PBS), and lysed using the extraction buffer provided in the assay kits. The cell suspensions were disrupted on ice and then centrifuged at 4 °C. The supernatants were collected for the determination of intracellular redox-related indices. Total protein concentration in each cell lysate was determined using a bicinchoninic acid (BCA) protein assay (Beyotime Biotech Inc.‌, Shanghai, China) and used for normalization.

The activities of superoxide dismutase (SOD) and catalase (CAT), as well as the content of glutathione (GSH) and MDA, were measured in rat serum and HOK cell lysate supernatants using commercial kits (Beijing Solarbio Science & Technology Co., Ltd., Beijing, China) according to the manufacturer’s protocols.

### 2.11. Enzyme-Linked Immunosorbent Assay (ELISA)

Serum samples and HOK cell culture supernatants were used to quantify inflammatory cytokines and repair-related factors using ELISA kits (Shanghai Youxuan Biotechnology Co., Ltd., Shanghai, China). For serum samples, blood was allowed to clot and then centrifuged to obtain serum. For cell experiments, culture supernatants were collected after treatment and centrifuged to remove cell debris before analysis. The analytes included TNF-α, IL-6, IL-1β, and epidermal growth factor (EGF). Standards and samples were added to precoated plates and incubated following the kit instructions. After washing, HRP-conjugated detection antibodies and substrate solution were applied, and absorbance was read at 450 nm using a microplate reader (SpectraMax iD3, Molecular Devices, LLC, San Jose, CA, USA). The concentrations of TNF-α, IL-6, IL-1β, and EGF were calculated from standard curves.

### 2.12. Histopathological Analysis

Three rats were randomly selected from each group for histological assessment. Ulcer tissues were fixed in 4% paraformaldehyde for 24 h, dehydrated through a graded ethanol series, embedded in paraffin, and sectioned at a thickness of 4 μm. For general morphological assessment, sections were stained with a hematoxylin and eosin (H&E) staining kit (Wuhan Servicebio Technology Co., Ltd., Wuhan, China). Histopathological evaluation focused on epithelial integrity, ulcer depth, inflammatory cell infiltration, edema, and granulation tissue formation. In addition, Masson’s trichrome staining was performed using a Masson staining kit (Wuhan Servicebio Technology Co., Ltd., Wuhan, China) to assess collagen deposition and fibrosis-related changes in the ulcer area. For H&E and Masson’s trichrome staining, one section per animal per staining method was obtained through the ulcer center. Each section underwent whole-slide scanning and examination. Images for presentation were randomly selected from the ulcer regions of the scanned sections. Images were captured and documented using an optical microscope (Nikon Eclipse E100, Nikon Corporation, Tokyo, Japan).

### 2.13. Immunofluorescence Staining Analysis

Paraffin sections were processed for immunofluorescence staining. After deparaffinization/rehydration and antigen retrieval, sections were blocked with normal serum and incubated overnight at 4 °C with primary antibodies against phosphorylated STAT3 (p-STAT3) and phosphorylated EGFR (p-EGFR). After washing, sections were incubated with species-appropriate fluorescent secondary antibodies and counterstained with 4′,6-diamidino-2-phenylindole (DAPI). Fluorescence images were acquired under identical exposure settings, and mean fluorescence intensity was quantified using image analysis software in a blinded manner.

### 2.14. Terminal Deoxynucleotidyl Transferase-Mediated dUTP Nick End Labeling (TUNEL) Assay

Apoptotic cells in ulcer tissues and HOK cells were detected using a TUNEL assay kit (Wuhan Servicebio Technology Co., Ltd., Wuhan, China). For ulcer tissues, paraffin sections were deparaffinized and rehydrated. After proteinase K treatment (37 °C for 20 min), sections were incubated with TUNEL reaction mixture in a humidified chamber at 37 °C for 1 h. HOK cells were seeded on coverslips or in culture plates, fixed after treatment, permeabilized, and then incubated with the TUNEL reaction mixture according to the manufacturer’s protocol. Nuclei were counterstained with DAPI. Images were captured using a fluorescence microscope (Nikon Eclipse C1, Nikon Corporation, Japan). The apoptotic index was calculated as:

Apoptotic index (%) = (TUNEL-positive nuclei/total nuclei) × 100.

For both tissue sections and cell samples, at least six randomly selected fields were analyzed for each sample.

### 2.15. Fluorescence Staining of Mitochondrial Membrane Potential (MMP), Intracellular Reactive Oxygen Species (ROS), and Lipid Droplets

After completion of the group-specific treatments described in Section 2.8, HOK cells were used for fluorescence staining to assess mitochondrial membrane potential, intracellular ROS, and lipid droplet accumulation.

For mitochondrial membrane potential detection, live HOK cells were incubated with 5,5′,6,6′-tetrachloro-1,1′,3,3′-tetraethylbenzimidazolylcarbocyanine iodide (JC-1) working solution from a JC-1 staining kit (Beijing Solarbio Science & Technology Co., Ltd., Beijing, China) according to the manufacturer’s instructions. After incubation, cells were washed with PBS, and fluorescence images were captured using a fluorescence microscope. The red/green fluorescence ratio was used to evaluate changes in mitochondrial membrane potential.

Intracellular ROS levels were detected using 2′,7′-dichlorodihydrofluorescein diacetate (DCFH-DA) (Beijing Solarbio Science & Technology Co., Ltd., Beijing, China). Briefly, live HOK cells were incubated with DCFH-DA working solution according to the manufacturer’s protocol. After incubation, cells were washed with PBS to remove excess probe, and fluorescence images were acquired using a fluorescence microscope. ROS accumulation was quantified based on fluorescence intensity.

For lipid droplet staining, HOK cells were fixed after treatment and incubated with BODIPY 493/503 staining solution (Beyotime Biotech Inc., Shanghai, China) according to the manufacturer’s instructions. After washing, fluorescence images were captured, and lipid droplet accumulation was evaluated based on fluorescence intensity or lipid droplet-positive area.

For all fluorescence staining assays, images were captured under consistent exposure settings within each experiment, and quantitative analysis was performed using ImageJ software.

### 2.16. Transwell Migration Assay and Wound Healing Assay

The migratory capacity of HOK cells was evaluated using Transwell chambers. After H_2_O_2_ exposure and subsequent drug intervention, cells were collected and resuspended in serum-free medium. The cell suspension was added to the upper chamber of Transwell inserts, while complete medium was added to the lower chamber as a chemoattractant. After incubation, non-migrated cells on the upper surface of the membrane were gently removed. Cells that migrated to the lower surface were fixed and stained with crystal violet solution (Beyotime Biotech Inc., Shanghai, China). Images were captured under a microscope, and migrated cells were counted in randomly selected fields.

A wound healing assay was performed to further assess HOK cell migration. Cells were seeded into culture plates and grown to near confluence. A straight scratch was generated in the cell monolayer using a sterile pipette tip, and detached cells were removed by washing with PBS. Cells were then treated according to the experimental grouping in serum-free medium. Images of the scratch area were captured at 0 h and 36 h under the same microscopic settings. The wound closure rate was calculated using ImageJ software.

### 2.17. Western Blot Analysis

After H_2_O_2_ exposure and subsequent drug intervention, HOK cells were collected and lysed in RIPA lysis buffer containing protease and phosphatase inhibitors. The lysates were centrifuged at 4 °C, and the supernatants were collected for protein extraction. Total protein concentration was determined using a BCA protein assay. Equal amounts of protein were separated by SDS-PAGE and transferred onto polyvinylidene difluoride membranes. After blocking, the membranes were incubated overnight at 4 °C with primary antibodies against STAT3 (1:1500), p-STAT3 (1:1500), EGFR (1:1500), p-EGFR (1:1000), Bax (1:5000), Bcl-2 (1:5000), and GAPDH (1:1000). After washing, the membranes were incubated with the corresponding secondary antibodies. Protein bands were visualized using an enhanced chemiluminescence substrate kit (Guangzhou Mutual Success Technology Co., Ltd., Guangzhou, China) and quantified using ImageJ software. GAPDH was used as the internal loading control. The relative phosphorylation levels of STAT3 and EGFR were expressed as p-STAT3/STAT3 and p-EGFR/EGFR, respectively. Bax and Bcl-2 expression levels were normalized to GAPDH.

### 2.18. Statistical Analysis

All data were expressed as mean ± standard deviation (SD). Ulcer area reduction percentages on days 3, 5, and 7 were analyzed using two-way repeated-measures ANOVA, with treatment group as the between-subject factor and time as the within-subject factor. Other comparisons among multiple groups were performed using one-way ANOVA followed by a suitable post hoc test. Non-normally distributed data were analyzed using a nonparametric test. A *p*-value < 0.05 was considered statistically significant. Statistical analyses were performed using GraphPad Prism 9.

## 3. Results

### 3.1. Preparation and Yield of YMKY Extract

YMKY extract was prepared for serum pharmacochemical analysis by aqueous decoction, concentration, and lyophilization. A total of 504.34 g of dried extract was obtained, corresponding to an extraction yield of 50.43% (*w*/*w*). The dried extract appeared as a blackish-brown powder and was used for serum constituent profiling. Among the serum-exposed prototype constituents identified from this analysis, EA was selected for subsequent pharmacological validation in the RAU model.

### 3.2. Identification of Serum-Exposed Prototype Constituents

**Figure 1:**
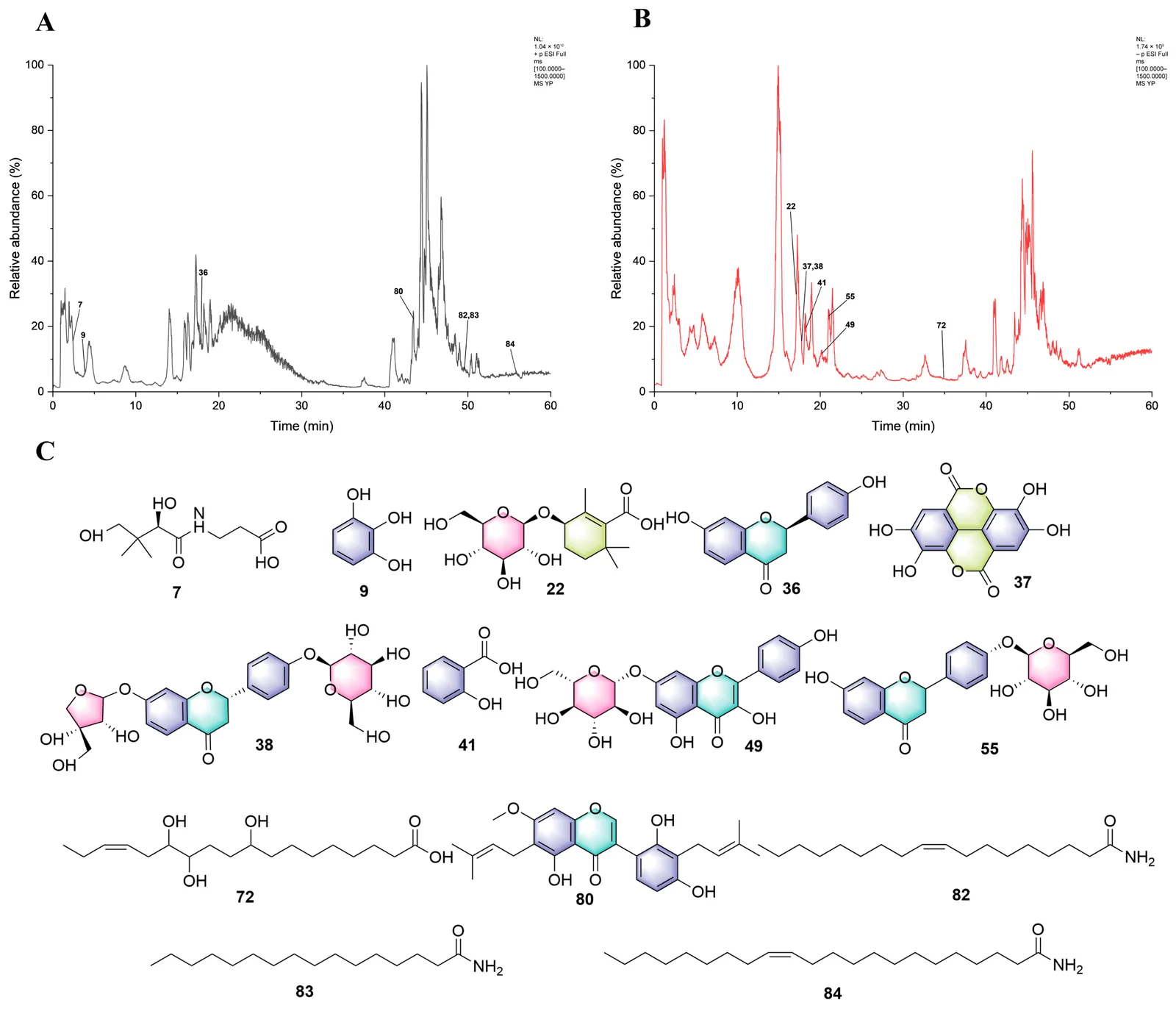
Serum pharmacochemical identification of prototype constituents absorbed after YMKY administration. (**A**) Total ion chromatogram of rat serum samples collected after YMKY administration in positive ESI mode. (**B**) Total ion chromatogram of rat serum samples collected after YMKY administration in negative ESI mode. Labeled peaks indicate prototype constituents detected in serum. (**C**) Chemical structures of the serum-exposed prototype constituents identified by UPLC-Q Exactive-Orbitrap HRMS. EA was annotated as peak 37 and selected for subsequent EA-centered mechanistic validation.

YMKY extract was analyzed by UPLC-Q Exactive-Orbitrap HRMS in both positive and negative ion modes. In total, 84 compounds were annotated based on accurate mass, MS/MS fragmentation patterns, and literature data (Figures S1 and S2 and Table S1), with an annotation confidence of >95%. Among them, 14 prototype constituents were detected in rat serum (Figure 1 and Table 1). EA was identified as Peak No. 37 and was retained as a candidate compound for subsequent target prediction, docking, and experimental validation.

### 3.3. Network Pharmacology Intersection and PPI Analysis

**Figure 2:**
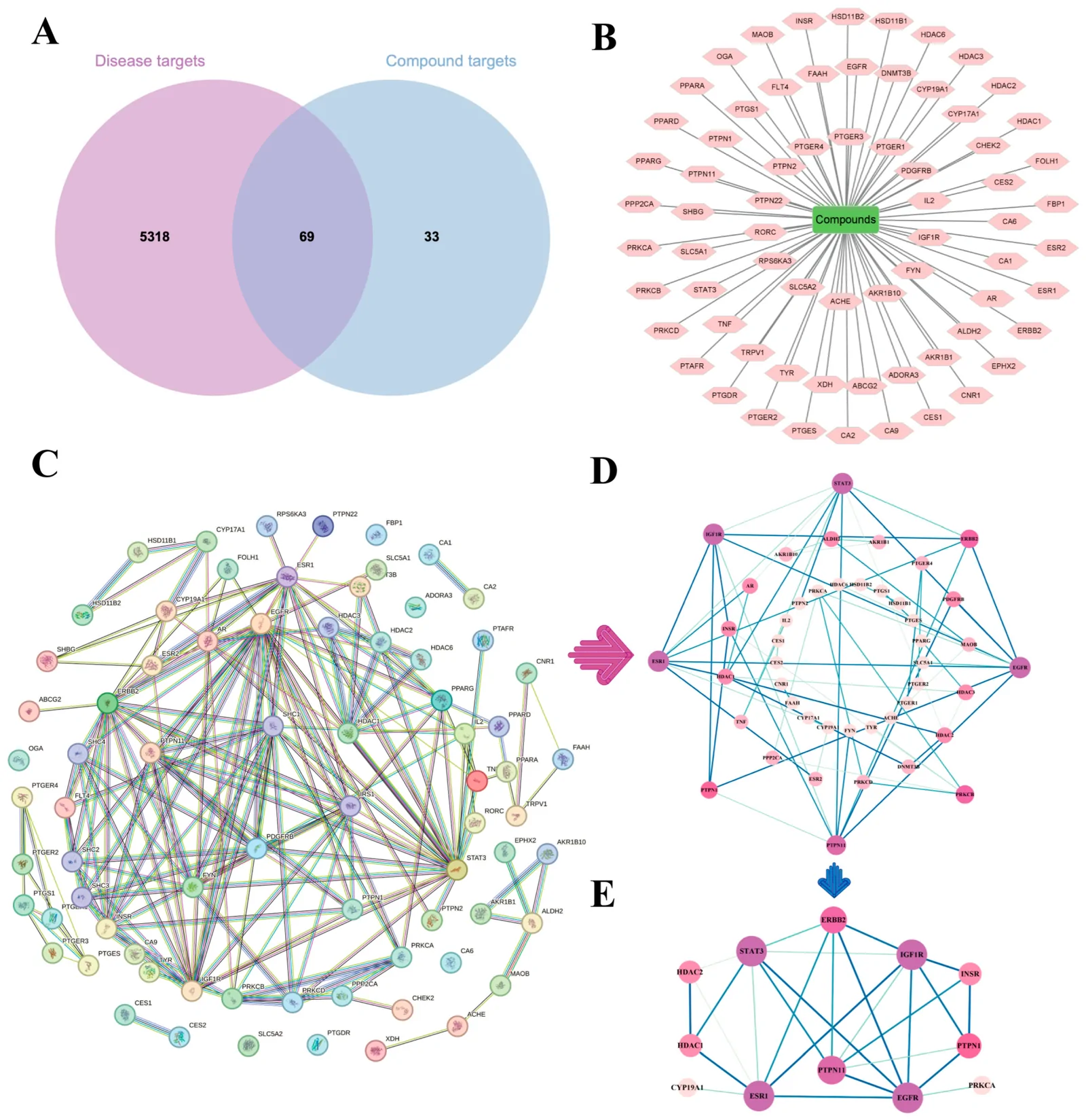
Network pharmacology and PPI analysis of serum-exposed YMKY constituents against RAU-related targets. (**A**) Venn diagram showing the intersection between predicted targets of serum-exposed prototype constituents and RAU-related genes. (**B**) Compound-target network constructed from the serum-exposed constituent screen. (**C**) STRING-based PPI network of the overlapping targets. (**D**) Cytoscape topological analysis of the PPI network. (**E**) Hub-target subnetwork identified by integrated topological ranking. STAT3 and EGFR were among the prioritized hub targets and were selected for subsequent docking and mechanistic validation.

As shown in Figure 2A, the intersection of the predicted target set for the serum-exposed prototype constituents and the RAU-related gene set yielded 69 shared targets. These targets were used to construct a PPI network for hub-target screening. Topological analysis identified STAT3 and EGFR as highly connected nodes (Figure 2D,E), suggesting that inflammatory signaling and epithelial repair may be key processes involved in the activity of the absorbed constituents.

The selection of STAT3 and EGFR was also biologically consistent with current evidence on oral mucosal injury and repair. STAT3 is involved in inflammatory transcriptional programs and has been implicated in oral mucosal wound healing. Recent evidence indicates that STAT3 acts as a hub transcription factor in rapid oral mucosal repair [12]. In an in vivo tongue wound model, IL-22-mediated STAT3 activation in keratinocytes promoted oral mucosal healing [13]. RAU is also associated with cytokine dysregulation, and a recent meta-analysis reported higher serum IL-6 levels in patients with RAU than in controls, supporting the relevance of STAT3-linked inflammatory signaling [40]. EGFR is a central regulator of epithelial homeostasis and wound closure. EGF/EGFR signaling promotes epithelial migration, proliferation, and re-epithelialization, whereas sustained EGFR inhibition can impair wound repair [41]. Thus, the identification of STAT3 and EGFR as hub targets provided a mechanistic basis for subsequent docking and experimental validation.

### 3.4. GO Enrichment Analysis

**Figure 3:**
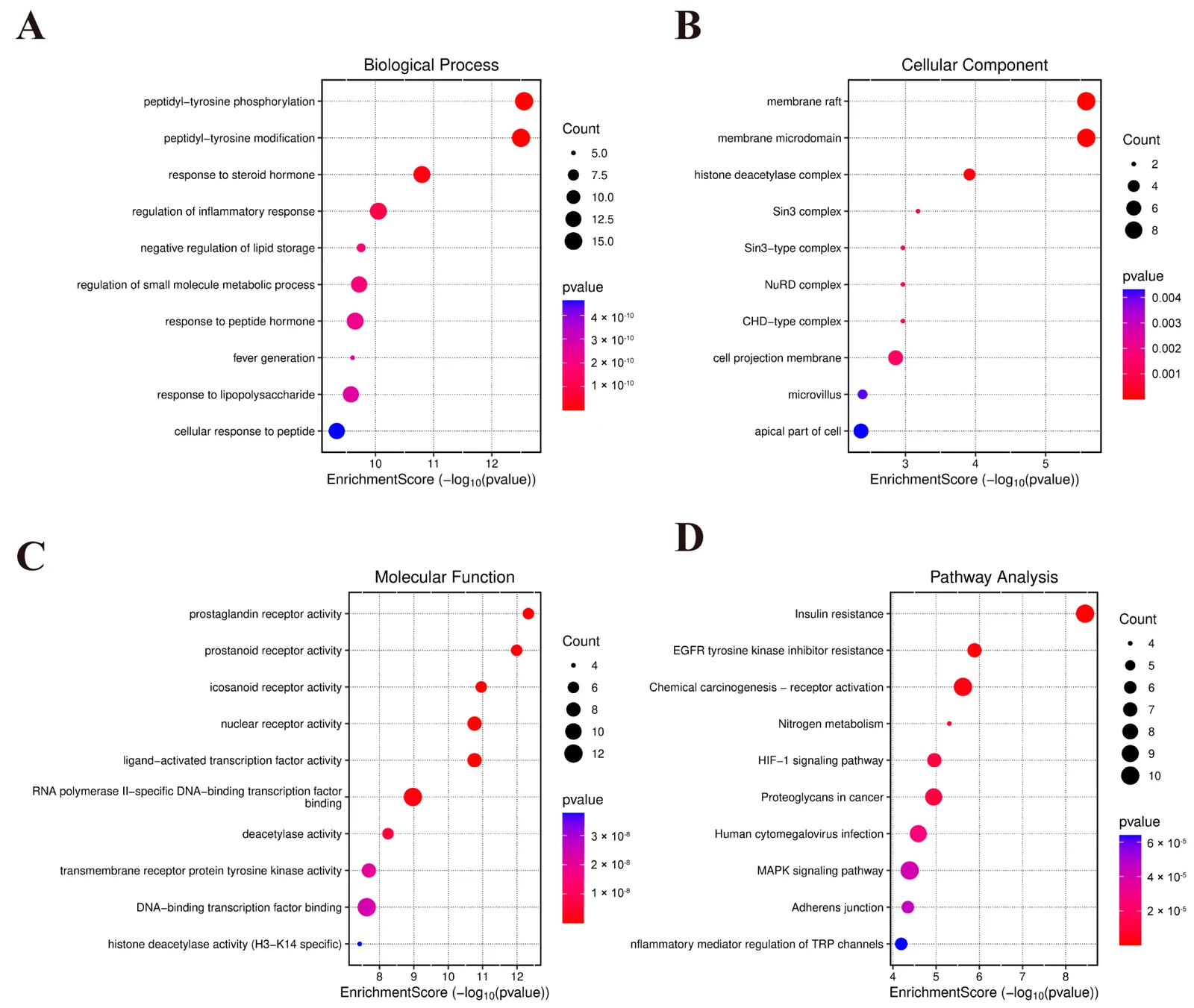
GO and KEGG enrichment overview of candidate targets. (**A**) GO BP enrichment results. (**B**) GO CC enrichment results. (**C**) GO MF enrichment results. (**D**) KEGG pathway enrichment results. In each dot plot, the *x*-axis represents the enrichment score (−log_10_ *p* value), dot size indicates the number of genes mapped to each term (Count), and dot color denotes the *p*-value.

To further clarify the biological relevance of the candidate targets identified by network pharmacology, GO enrichment analysis was performed, and the results are summarized in Figure 3A–C. In the Biological Process (BP) category (Figure 3A), the enriched terms were mainly related to tyrosine phosphorylation-dependent signaling, regulation of inflammatory responses, and responses to external stimuli, indicating that the candidate targets may participate in inflammatory signal transduction and stress-responsive pathways. In the Cellular Component (CC) category (Figure 3B), the enriched terms were concentrated in membrane microdomains (e.g., membrane rafts/microdomains) and nuclear chromatin-associated regulatory complexes, suggesting a spatial organization that involves both membrane-proximal signaling platforms and nuclear transcriptional regulation. In the Molecular Function (MF) category (Figure 3C), the candidate targets were enriched in functions associated with lipid mediator receptor activity, nuclear receptor-related transcriptional regulation, and transmembrane receptor protein tyrosine kinase activity, together implying coordinated modulation of inflammatory mediator sensing, transcriptional control, and receptor-driven signaling.

To connect enriched terms with specific genes and avoid redundant interpretation, term-gene association networks were examined (Figure S3A–C). These cnetplots highlighted a set of genes repeatedly mapped to multiple enriched terms, including receptor- and kinase-related nodes and transcriptional regulators, supporting their central positions across distinct functional layers. The similarity networks (emapplots) further indicated that enriched terms could be organized into several highly connected clusters, facilitating module-level interpretation (Figure S4A–C).

### 3.5. KEGG Pathway Enrichment Analysis

KEGG pathway enrichment analysis provided pathway-level context for these functional annotations (Figure 3D). The enriched pathways primarily converged on receptor-linked signaling and downstream cascades, inflammation- and stress-response pathways, and cell junction-related processes, consistent with a multi-target regulatory pattern implicated in ulcer-associated inflammation and mucosal recovery. Several KEGG entries contain broad “disease” labels (e.g., cancer- or infection-related pathways), which frequently emerge in enrichment analyses due to shared core signaling nodes; therefore, these pathway names were interpreted as reflecting common signaling modules rather than disease specificity. The distribution of mapped targets within representative pathways was visualized using pathview (Figures S5–S9), including the MAPK signaling pathway, HIF-1 signaling pathway, adherens junction pathway, JAK-STAT signaling pathway, and inflammatory mediator regulation of transient receptor potential channels pathway. Together, the GO and KEGG results support the network-level prioritization and provide a functional framework for subsequent validation experiments.

### 3.6. Molecular Docking Validation of Serum-Absorbed Constituents Against STAT3 and EGFR

To provide structural support for the hub targets identified by network analysis, the serum-absorbed prototype constituents detected by UPLC-Q Exactive-Orbitrap HRMS were docked to STAT3 and EGFR. Docking was performed using AutoDock Vina, with grid boxes defined around the reported binding-site residues of STAT3 (ASP570, ASP566, ARG335, PRO333, ASP334, and HIS332) and EGFR (LEU679, ARG681, LEU754, ALA743, MET742, and VAL745). Binding affinities were calculated from five independent docking runs and are presented as mean ± SD. As summarized in Table S2, the predicted binding energies ranged from −3.98 to −8.10 kcal/mol for STAT3 and from −4.14 to −8.06 kcal/mol for EGFR. Among the tested constituents, EA showed the most favorable binding toward both targets, with binding energies of −8.10 ± 0.27 kcal/mol for STAT3 and −8.06 ± 0.19 kcal/mol for EGFR. The next best-scoring ligands were liquiritigenin-7-*O*-β-D-apiofuranosyl-4′-*O*-β-D-glucopyranoside (Peak No. 38; STAT3: −7.80 ± 0.26 kcal/mol; EGFR: −8.02 ± 0.16 kcal/mol) and kaempferol-7-*O*-β-D-glucopyranoside (Peak No. 49; STAT3: −7.62 ± 0.22 kcal/mol; EGFR: −7.96 ± 0.29 kcal/mol). Although these glycosylated flavonoids also showed strong docking scores, their lower ligand efficiency was consistent with their larger molecular size.

**Figure 4:**
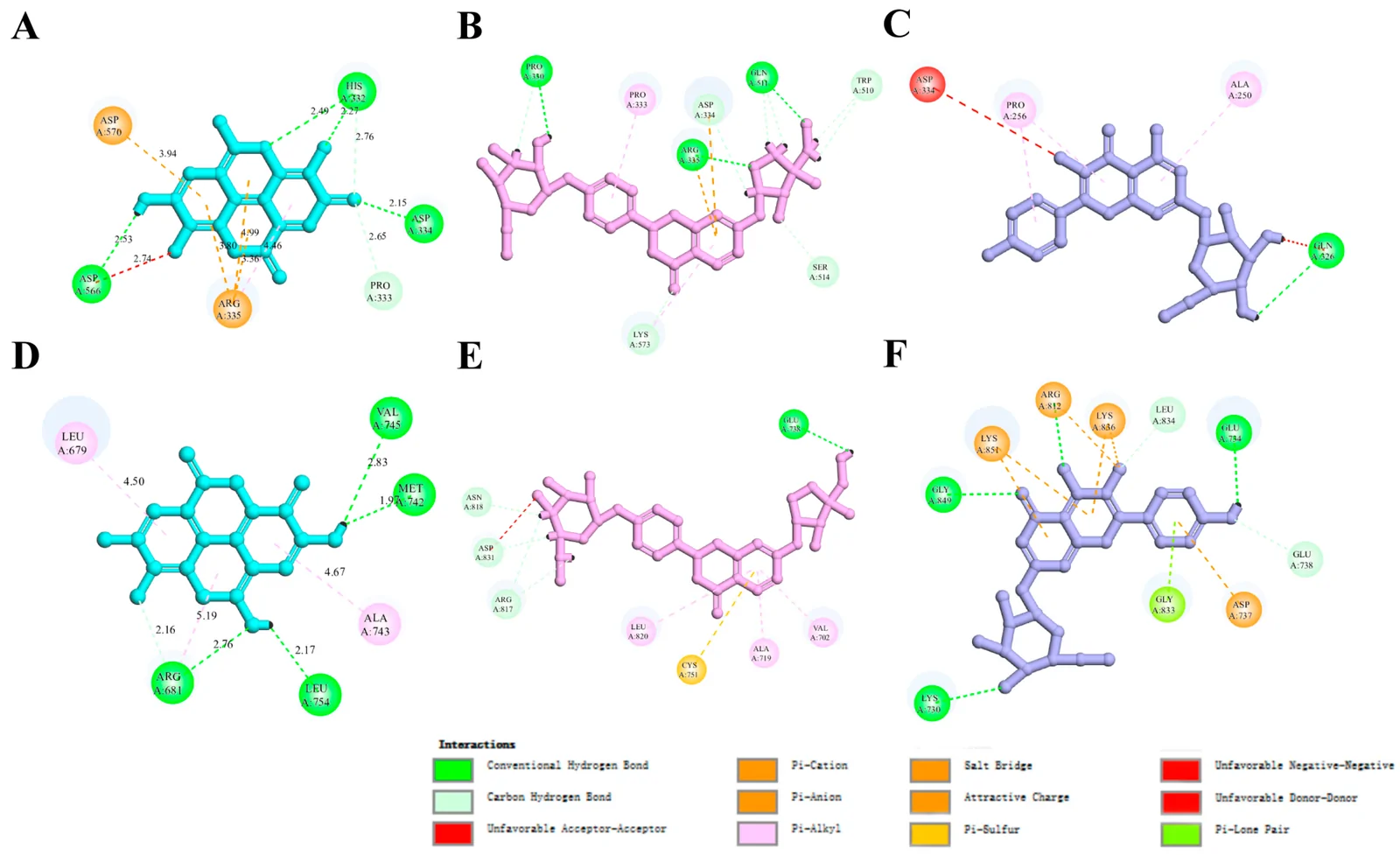
Molecular docking of the top-ranked serum-absorbed constituents with STAT3 and EGFR. (**A**–**C**) Two-dimensional interaction diagrams of STAT3 docked with the three candidates showing the most favorable predicted binding affinities: EA (**A**), compound 38 (**B**), and compound 49 (**C**). (**D**–**F**) Two-dimensional interaction diagrams of EGFR docked with EA (**D**), compound 38 (**E**), and compound 49 (**F**). Interaction types are indicated by the color-coded legend.

The binding modes of the three top-ranked ligands are shown in Figure 4. For STAT3, EA (Figure 4A) occupied the predefined binding pocket and formed hydrogen bonds with ASP566, HIS332, and ASP334, together with an electrostatic interaction involving ARG335. Liquiritigenin-7-O-β-D-apiofuranosyl-4′-*O*-β-D-glucopyranoside (Figure 4B) adopted a more extended conformation and contacted additional surrounding residues. Kaempferol-7-*O*-β-D-glucopyranoside (Figure 4C) also occupied the same cavity but showed a distinct interaction pattern. For EGFR (Figure 4D–F), EA (Figure 4D) was positioned in the kinase-domain pocket and interacted with residues around LEU679, ARG681, MET742, ALA743, VAL745, and LEU754. The other two ligands (Figure 4E,F) showed broader contact patterns around the binding site.

**Figure 5:**
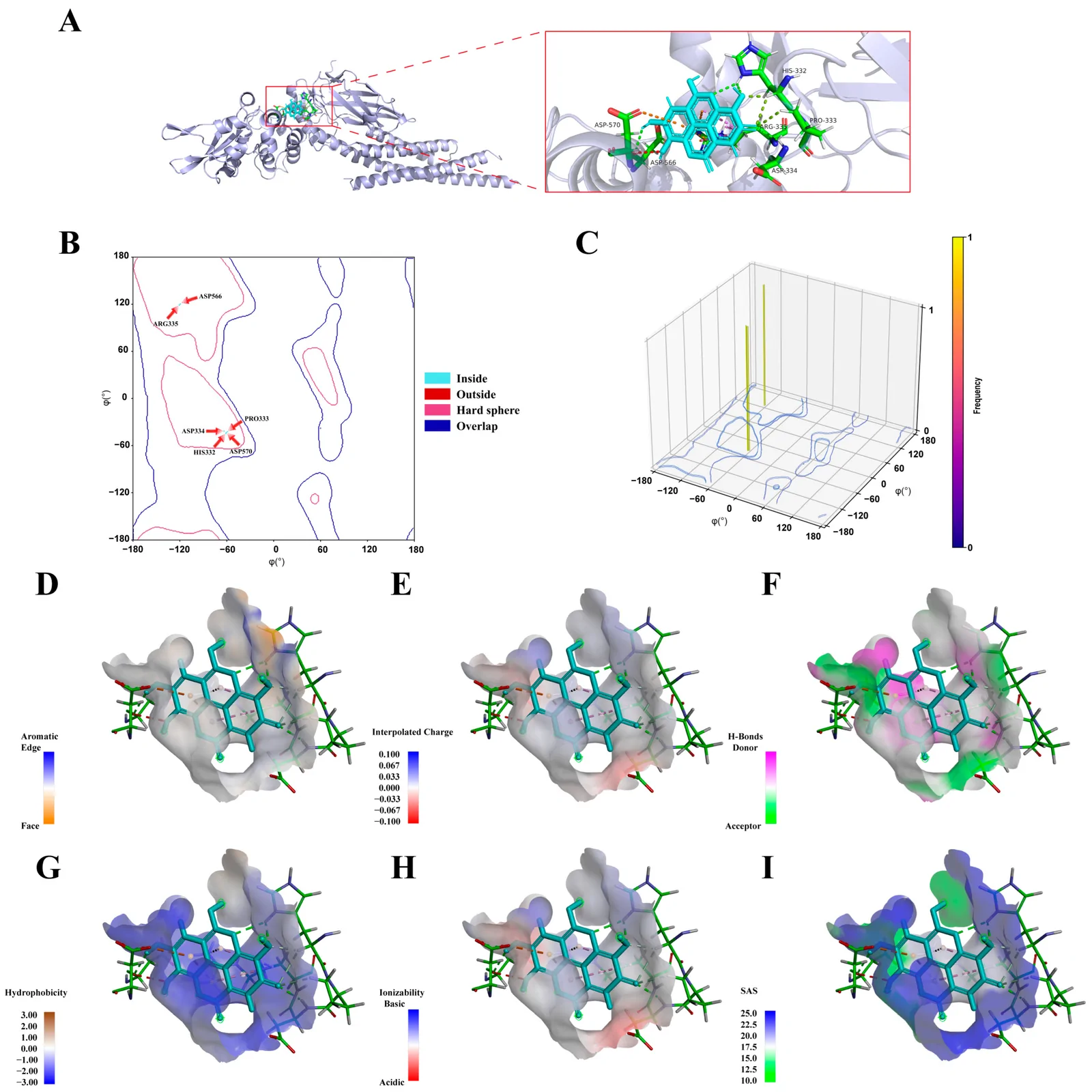
Structural characterization of the docked complex between EA and STAT3. (**A**) Representative docking pose of EA within the STAT3 binding pocket. (**B**,**C**) Ramachandran plot analysis of the STAT3 complex shown in two-dimensional (**B**) and three-dimensional (**C**) formats. (**D**–**I**) Surface-based interaction profiling of EA at the STAT3 active site, including aromatic surface (**D**), interpolated charge surface (**E**), hydrogen-bond surface (**F**), hydrophobicity surface (**G**), ionizability surface (**H**), and solvent-accessible surface (**I**).

Because EA ranked highest against both STAT3 and EGFR, its binding poses were further examined in detail. In the EA-STAT3 complex (Figure 5A), EA was located within the active-site region and contacted ASP570, ASP566, ARG335, PRO333, ASP334, and HIS332. Ramachandran plots (Figure 5B,C) showed that the highlighted residues were located in allowed conformational regions. Surface analysis further indicated that EA was accommodated by the STAT3 pocket through aromatic, electrostatic, hydrogen-bonding, hydrophobic, and ionizability-related complementarity (Figure 5D–I).

**Figure 6:**
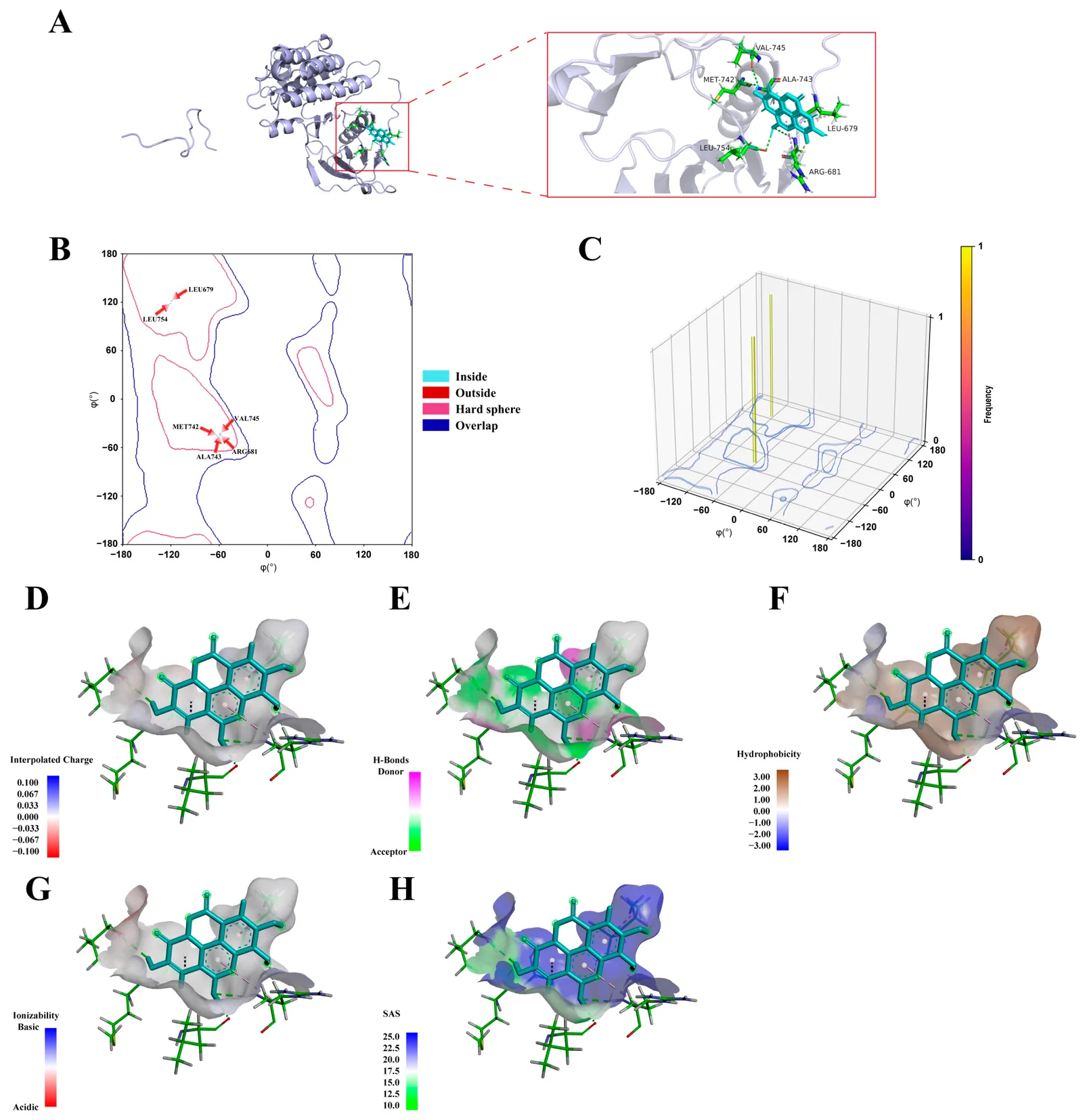
Structural characterization of the docked complex between EA and EGFR. (**A**) Representative docking pose of EA within the EGFR kinase-domain pocket. (**B**,**C**) Ramachandran plot analysis of the EGFR complex shown in two-dimensional (**B**) and three-dimensional (**C**) formats. (**D**–**H**) Surface-based interaction profiling of EA at the EGFR active site, including interpolated charge surface (**D**), hydrogen-bond surface (**E**), hydrophobicity surface (**F**), ionizability surface (**G**), and solvent-accessible surface (**H**).

A similar analysis was performed for the EA-EGFR complex (Figure 6). EA occupied the EGFR active pocket (Figure 6A) and contacted LEU679, ARG681, MET742, ALA743, VAL745, and LEU754. Ramachandran plots (Figure 6B,C) supported the local stereochemical quality of the modeled complex. Surface representations suggested that the EA-EGFR interaction was mainly supported by electrostatic complementarity, hydrogen-bonding potential, and hydrophobic fitting within the kinase pocket (Figure 6D–H).

Together, these docking results identified EA as the leading serum-absorbed candidate for STAT3 and EGFR binding and provided the basis for subsequent MD simulation and experimental validation.

### 3.7. Drug-Likeness and ADMET Predictions

The serum-absorbed prototype constituents exhibited overall acceptable drug-likeness and ADMET-related properties (Tables S3 and S4). Based on Lipinski’s rule of five, most compounds showed zero violations, whereas four compounds displayed at least one violation, with compound 38 and compound 49 showing the highest numbers of violations (Table S3). ADMET prediction further suggested that the majority of compounds had high predicted gastrointestinal (GI) absorption, while a subset showed low GI absorption, which was generally consistent with their physicochemical features (Table S3). In addition, the predicted absorption- and disposition-related parameters (including Caco-2 permeability, human intestinal absorption (HIA), plasma protein binding (PPB), water solubility, and P-glycoprotein inhibition) varied across compounds, reflecting heterogeneity in their pharmacokinetic behaviors (Table S4). Detailed compound-by-compound readouts are provided in the Supplementary Information.

### 3.8. MD Simulation of EA-STAT3 and EA-EGFR Complexes

**Figure 7:**
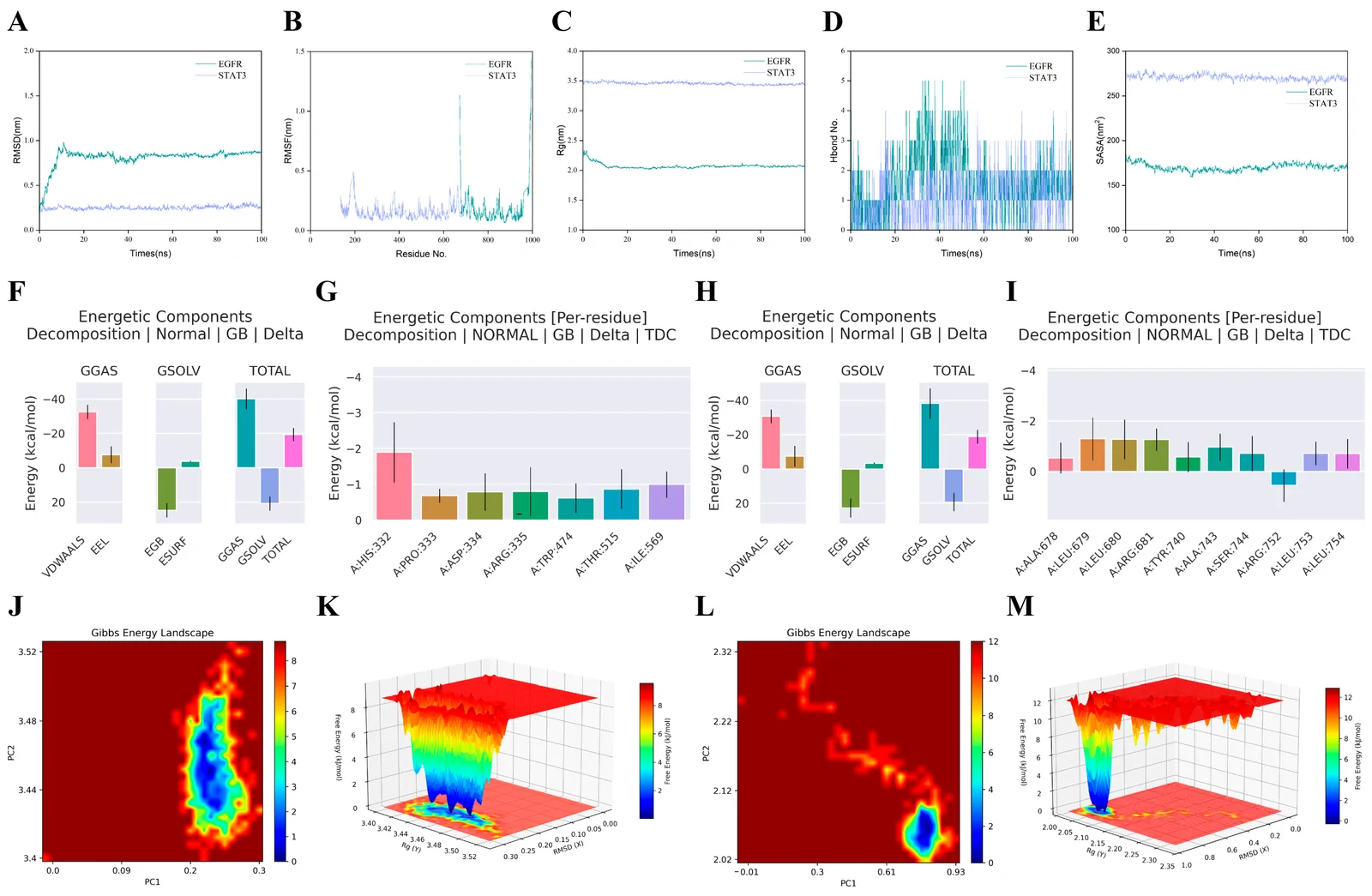
MD trajectory analyses and binding free energy decomposition for EA bound to STAT3 and EGFR. (**A**) RMSD profiles of the EA-STAT3 and EA-EGFR complexes during the 100 ns simulation, (**B**) RMSF of protein residues for both complexes, (**C**) Rg trajectories for both complexes, (**D**) Time-dependent changes in the number of H-bonds between EA and each protein, (**E**) SASA trajectories for both complexes, (**F**) MM/GBSA energy component decomposition for the EA-STAT3 complex, (**G**) Per-residue binding free energy contributions of key residues in the EA-STAT3 complex, (**H**) MM/GBSA energy component decomposition for the EA-EGFR complex, (**I**) Per-residue binding free energy contributions of key residues in the EA-EGFR complex, (**J**,**K**) 2D (**J**) and 3D (**K**) representations of the FEL of the EA-STAT3 complex derived from the MD trajectory. (**L**,**M**) 2D (**L**) and 3D (**M**) representations of the FEL of the EA-EGFR complex derived from the MD trajectory.

To further evaluate the docking poses of EA with STAT3 and EGFR, 100 ns MD simulations were performed for the EA-STAT3 and EA-EGFR complexes. The trajectories were assessed using RMSD, RMSF, radius of gyration (Rg), hydrogen-bond number, and solvent-accessible surface area (SASA) (Figure 7A–E). Free energy landscape (FEL) analysis was also used to identify the main low-energy conformational states sampled during the simulations (Figure 7J–M).

For the EA-STAT3 complex, the RMSD profile showed limited fluctuation during the 100 ns simulation and reached 0.2389 nm at the final frame (Figure 7A). RMSF analysis showed that most residues had low mobility (RMSF < 0.6 nm), whereas residues 192–198 showed relatively higher flexibility (Figure 7B). The Rg values remained within a narrow range of 3.4–3.5 nm, with a final value of 3.45096 nm, indicating that the complex maintained its overall compactness (Figure 7C). The number of protein-ligand hydrogen bonds ranged from 0 to 4 during the simulation (Figure 7D). SASA remained approximately 270 nm^2^, suggesting limited change in solvent exposure (Figure 7E). The FEL showed a defined low-energy basin for the EA-STAT3 complex, consistent with convergence toward a relatively stable conformational state (Figure 7J,K).

For the EA-EGFR complex, the RMSD curve showed an initial adjustment during the first approximately 10 ns and then became more stable, with a final RMSD of 0.88519 nm (Figure 7A). RMSF analysis indicated that most residues fluctuated within 1.5 nm, while residues 672–676 and 987–996 showed higher local mobility (Figure 7B). The Rg values remained between 2.0 and 2.4 nm and converged to 2.06738 nm at the final frame (Figure 7C). The number of hydrogen bonds fluctuated between 0 and 5 (Figure 7D), and SASA remained close to 170 nm^2^ (Figure 7E). The FEL contained distinct low-energy basins, indicating energetically preferred ligand-bound conformations during the simulation (Figure 7L,M).

The residues contributing to binding energy overlapped with the docking-grid regions, including HIS332, PRO333, ASP334, and ARG335 in STAT3, and LEU679, ARG681, ALA743, and LEU754 in EGFR. This overlap indicates that the MD-stabilized poses remained within the intended binding pockets rather than shifting to unrelated surface regions.

### 3.9. MM/GBSA Binding Free Energy and Residue Contributions

MM/GBSA analysis showed negative total binding free energies for both EA-protein complexes. The EA-STAT3 complex had a total binding free energy of −19.42 ± 3.84 kcal/mol (Figure 7F). Energy decomposition showed that van der Waals and electrostatic interactions contributed favorably to binding, whereas polar solvation opposed complex formation. The non-polar solvation term made a smaller favorable contribution, resulting in an overall negative ΔG (Figure 7F). Per-residue decomposition identified HIS332, PRO333, ASP334, ARG335, TRP474, THR515, and ILE569 as major contributors to EA binding (Figure 7G). The EA-EGFR complex showed a comparable total binding free energy of −18.96 ± 4.06 kcal/mol (Figure 7H). As observed for EA-STAT3, the favorable van der Waals and electrostatic terms were partly offset by an unfavorable polar solvation term (Figure 7H). Residue-level analysis identified ALA678, LEU679, LEU680, ARG681, TYR740, ALA743, SER744, LEU753, and LEU754 as key energetic contributors (Figure 7I).

Together with the MD trajectory analyses, the MM/GBSA results suggest that EA maintained energetically favorable interactions with STAT3 and EGFR throughout the 100 ns simulations, supporting the predicted stability of the docked noncovalent complexes. These findings provide computational support for EA-protein interactions.

### 3.10. DFT Analysis of EA

**Figure 8:**
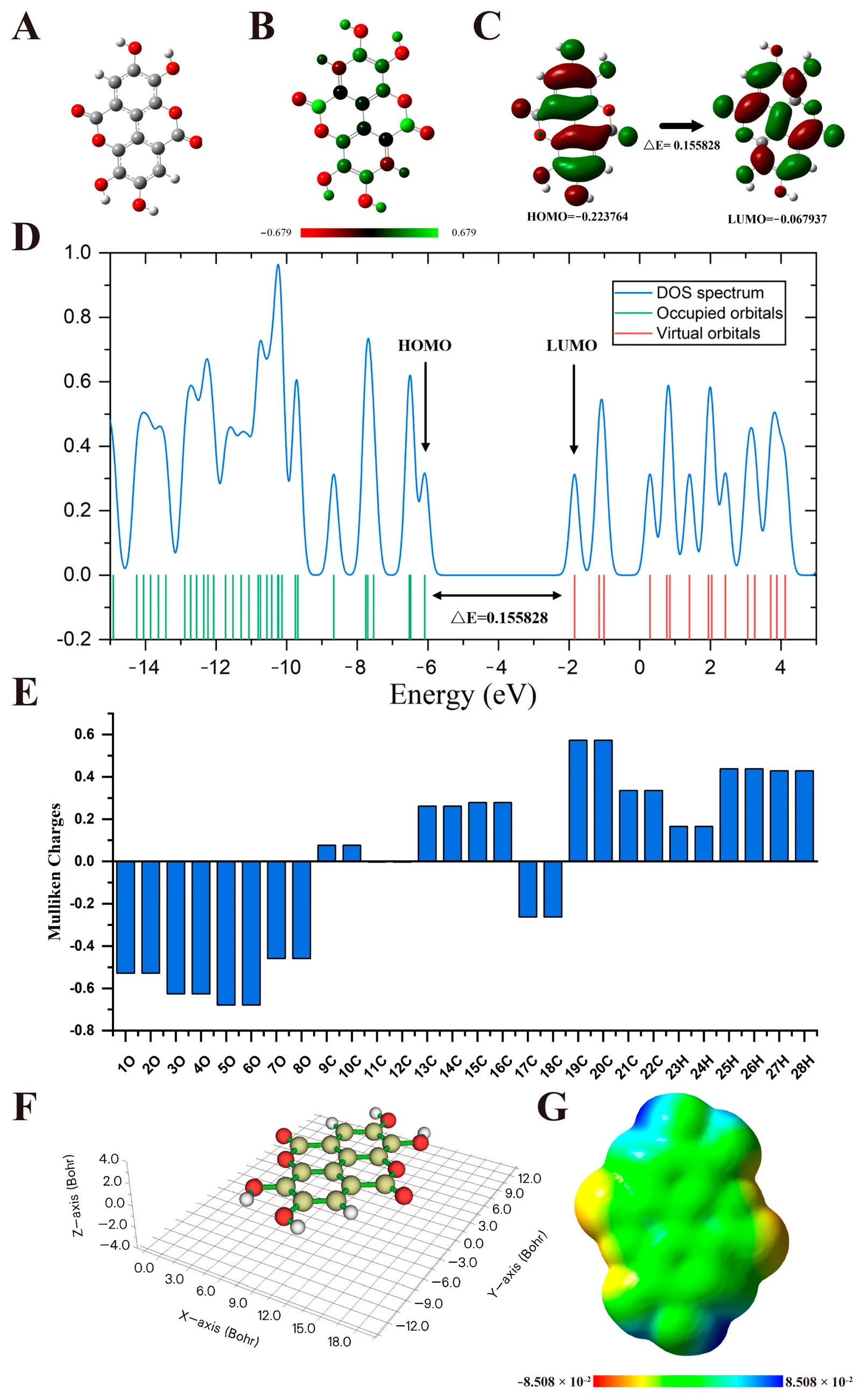
Density functional theory studies of EA. (**A**) Optimized structure of EA, (**B**) Color-coded representation of Mulliken atomic charges, (**C**) Bar graph showing atomic charges, (**D**) DOS spectra of EA, (**E**) HOMO-LUMO images of EA, (**F**) Critical points and topological paths of EA, and (**G**) MEP surface of EA.

DFT calculations were performed to characterize the intrinsic electronic structure of EA and to provide a physicochemical basis for interpreting its predicted interactions with STAT3 and EGFR (Figure 8). Geometry optimization yielded a minimum-energy conformation with a conjugated polyphenolic scaffold (Figure 8A). Mulliken population analysis showed that the oxygen-containing groups of EA carried relatively negative charges, whereas hydrogen atoms and selected carbon atoms were more positive (Figure 8B,C). This uneven charge distribution is compatible with the hydrogen-bond donor and acceptor properties of EA and provides a chemical basis for the polar contacts predicted in the docking poses.

The calculated HOMO and LUMO energies were −0.223764 and −0.067937 Hartree, with a HOMO-LUMO energy gap of 0.155827 Hartree (Figure 8D,E). The HOMO and LUMO densities were mainly localized around the conjugated aromatic and oxygen-containing regions, indicating that these moieties may be chemically relevant to the interaction propensity of EA. The MEP surface likewise showed electron-rich regions around oxygen atoms and electron-poor regions around hydrogen-bearing sites (Figure 8G).

Notably, these DFT-derived electronic descriptors do not establish specific molecular recognition of STAT3 or EGFR. Rather, the observed correspondence between the electron-rich regions of EA and the polar or hydrogen-bonding contacts predicted in the docking models provides molecular-level support for the chemical plausibility of the proposed interaction modes. The target specificity and stability of the EA-STAT3 and EA-EGFR complexes were evaluated separately by molecular docking, MD trajectory analysis, and MM/GBSA calculations.

### 3.11. EA Accelerates Macroscopic Ulcer Closure

**Figure 9:**
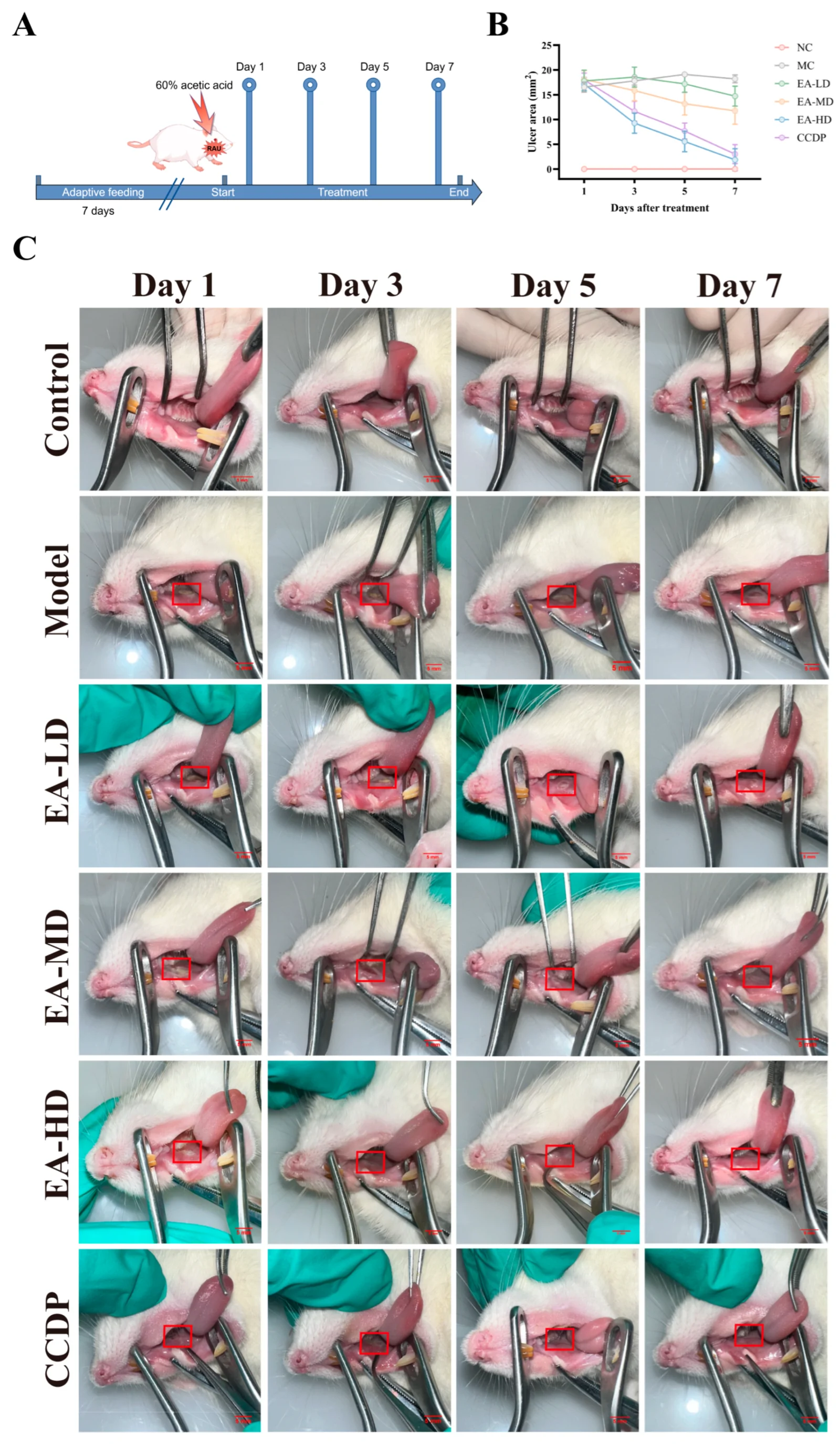
EA accelerates macroscopic healing of recurrent aphthous ulcers in rats. (**A**) Schematic timeline of RAU induction, EA intervention, and sample collection. (**B**) Quantification of ulcer area over time (days 1, 3, 5, and 7) based on ImageJ analysis. Data are presented as mean ± SD (*n* = 6). (**C**) Representative intraoral photographs of ulcers from each group on days 1, 3, 5, and 7 after modeling.

As illustrated in the experimental timeline (Figure 9A), RAU was induced by acetic acid application, and animals then received vehicle, CCDP, EA-LD (10 mg/kg/day), EA-MD (25 mg/kg/day), or EA-HD (50 mg/kg/day) for 7 consecutive days. Macroscopic ulcer healing was evaluated on days 1, 3, 5, and 7 after model establishment. Representative intraoral photographs showed persistent ulceration in the model group, whereas EA treatment promoted progressive lesion closure, most prominently in the EA-HD group (Figure 9C).

ImageJ-based quantification confirmed that ulcer areas were comparable among the modeled groups on day 1, ranging from 16.54 ± 1.02 to 18.09 ± 1.77 mm^2^ (Figure 9B). In the model group, ulcer area remained high throughout the observation period, increasing from 16.54 ± 1.02 mm^2^ on day 1 to 18.21 ± 0.78 mm^2^ on day 7. EA treatment reduced ulcer area in a dose-dependent manner. By day 7, the residual ulcer area decreased to 14.73 ± 2.00 mm^2^ in the EA-LD group, 11.76 ± 2.71 mm^2^ in the EA-MD group, and 1.87 ± 2.22 mm^2^ in the EA-HD group. The CCDP group showed a similar healing trend, with a day 7 ulcer area of 3.03 ± 1.91 mm^2^. These findings indicate that EA, particularly at 50 mg/kg/day, markedly accelerated macroscopic ulcer closure in RAU rats.

Body weight and food intake were monitored to evaluate the general condition of the animals (Figures S10 and S11). The model group showed a continuous decline in body weight, from 373.07 ± 9.48 g on day 1 to 330.48 ± 9.31 g on day 7. EA treatment mitigated this loss, especially in the EA-HD group, in which body weight was largely maintained from 372.56 ± 7.61 g to 367.64 ± 10.32 g over the same period. Food intake also recovered more clearly in the EA-HD group, increasing from 12.61 g on day 1 to 104.43 g on day 7, compared with 35.67 g in the model group on day 7.

### 3.12. EA Improves Histopathology and Collagen Deposition

**Figure 10:**
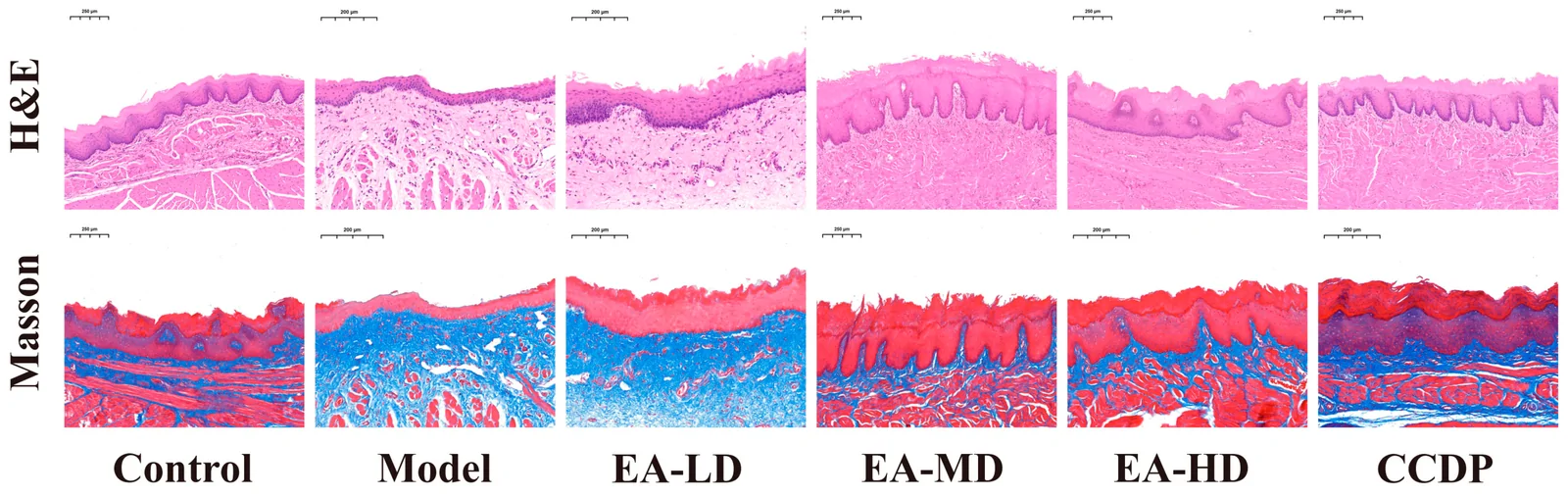
Representative histological images of ulcer tissues stained with H&E and Masson’s trichrome (*n* = 3).

To determine whether macroscopic ulcer closure was accompanied by tissue-level repair, ulcer tissues were examined by H&E and Masson’s trichrome staining (Figure 10). In the control group, the oral mucosa showed an intact epithelial layer with regular tissue architecture. In contrast, the model group exhibited epithelial disruption, inflammatory-cell infiltration, and loose stromal organization, indicating persistent mucosal injury. EA treatment alleviated these pathological changes in a dose-dependent manner. The EA-LD group showed partial epithelial recovery, whereas the EA-MD and EA-HD groups displayed more continuous epithelial coverage, reduced inflammatory infiltration, and improved mucosal organization. The histological appearance of the EA-HD group was close to that of the CCDP group, consistent with the macroscopic healing results.

Masson’s trichrome staining further showed marked disruption of collagen organization in the model group, with loose and irregular subepithelial fibers. After EA treatment, collagen deposition became denser and more organized, especially in the EA-MD and EA-HD groups. The EA-HD group showed a more continuous collagen network beneath the repaired epithelium, while the CCDP group exhibited a comparable pattern of matrix restoration. These findings indicate that EA promoted epithelial repair and extracellular matrix remodeling in RAU lesions.

### 3.13. EA Restores Redox Balance

**Figure 11:**
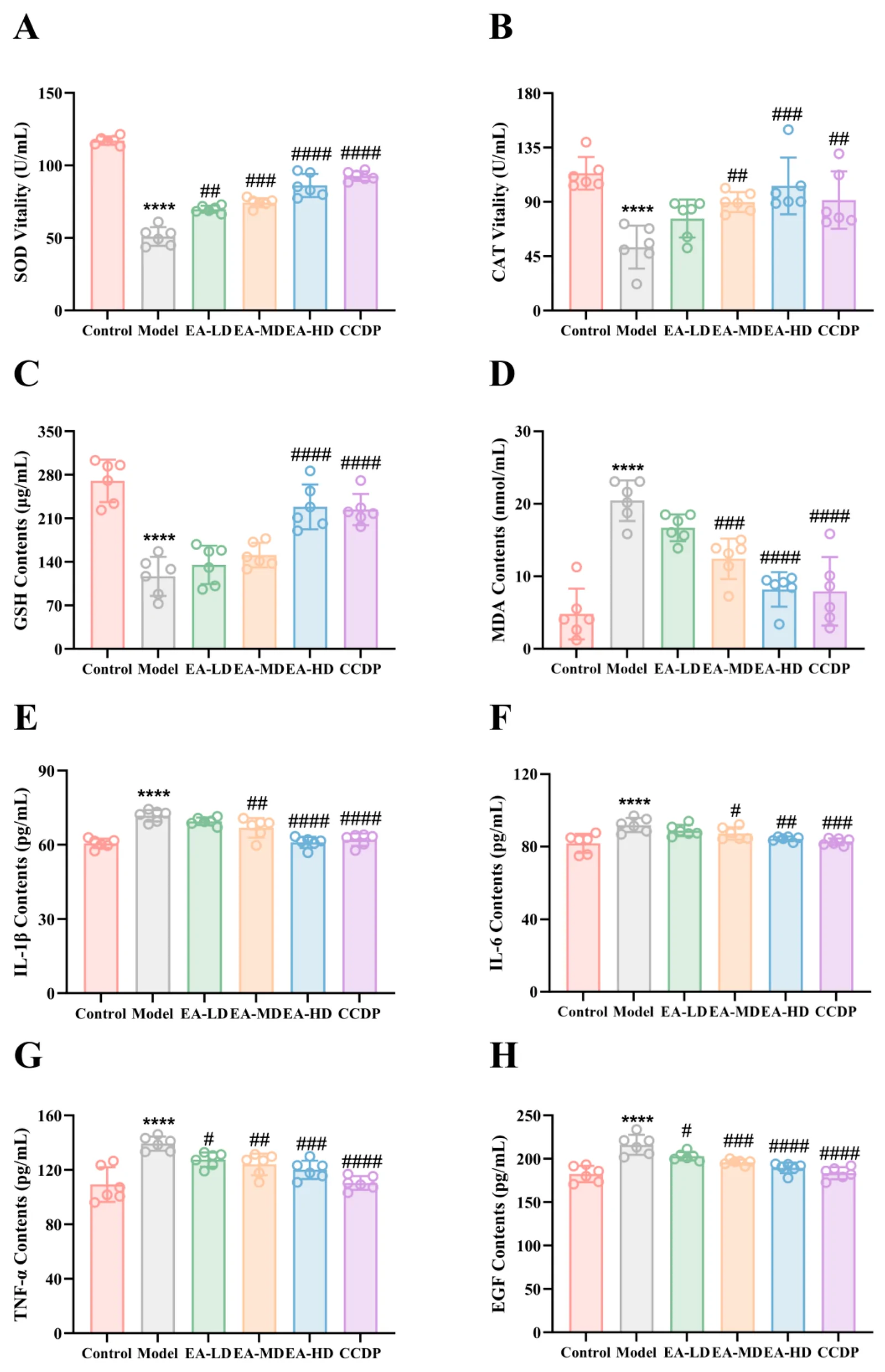
EA restores systemic redox homeostasis and modulates circulating inflammatory mediators in RAU rats. (**A**) SOD activity, (**B**) CAT activity, (**C**) GSH level, (**D**) MDA level, (**E**) IL-1β level, (**F**) IL-6 level, (**G**) TNF-α level, and (**H**) EGF level. Data are presented as mean ± SD (*n* = 6). Statistical significance is indicated in the graph. **** *p* < 0.0001 vs. the control group; ^#^ *p* < 0.05, ^##^ *p* < 0.01, ^###^ *p* < 0.001, and ^####^ *p* < 0.0001 vs. the model group.

Serum redox indices were measured to determine whether EA alleviated oxidative stress in RAU rats (Figure 11A–D). Compared with the control group, the model group showed marked decreases in SOD and CAT activities and GSH levels, accompanied by increased MDA content, indicating impaired antioxidant defense and enhanced lipid peroxidation. EA treatment reversed these changes to varying degrees. SOD and CAT activities, as well as GSH levels, were increased in the EA-treated groups compared with the model group, whereas MDA content was reduced. The recovery was most evident in the EA-HD group, which showed a profile close to that of the CCDP group. These results indicate that EA restored redox homeostasis in RAU rats.

### 3.14. EA Attenuates Inflammation and Repair-Related Mediator Dysregulation

Serum inflammatory cytokines and EGF were measured to evaluate whether EA modulated the inflammatory-repair milieu in RAU rats (Figure 11E–H). Compared with the control group, the model group showed elevated IL-1β, IL-6, TNF-α, and EGF levels, indicating activation of inflammatory and injury-associated repair responses after RAU induction. EA treatment reduced the levels of IL-1β, IL-6, and TNF-α, with the most pronounced decreases observed in the EA-HD group. EGF was also reduced after EA treatment, suggesting partial normalization of the stress-associated EGF response. These findings show that EA attenuated systemic inflammatory activation and helped rebalance the inflammatory-repair response in RAU rats.

### 3.15. EA Modulates STAT3/EGFR Signaling and Reduces Apoptosis in Ulcer Tissues

**Figure 12:**
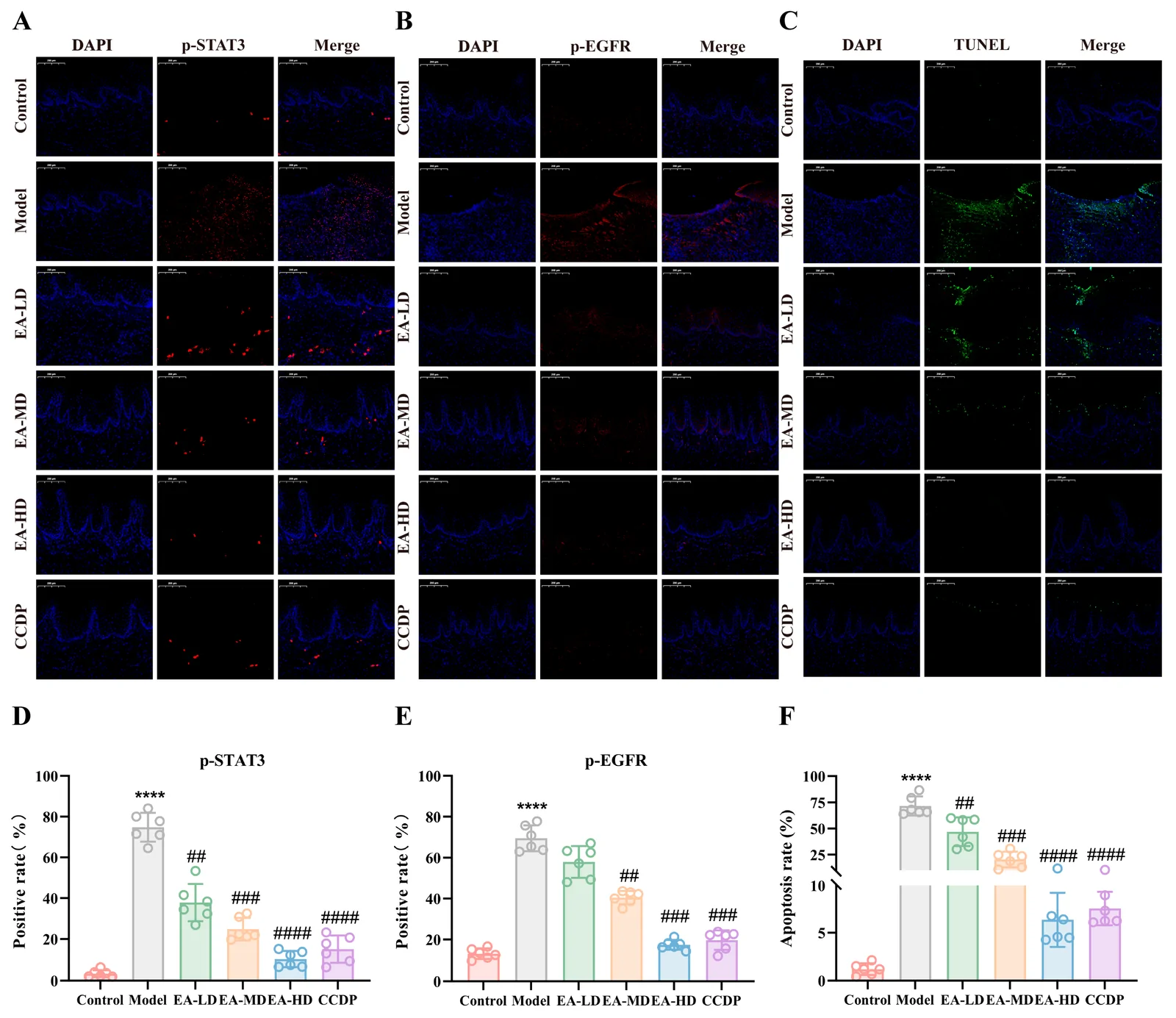
EA normalizes STAT3/EGFR signaling activation and reduces apoptosis in ulcer tissues. Representative immunofluorescence images of ulcer tissues. (**A**) p-STAT3 staining, (**B**) p-EGFR staining, (**C**) TUNEL staining for apoptotic cells. Nuclei were counterstained with DAPI. (**D**–**F**) Quantification of p-STAT3 positivity (**D**), p-EGFR positivity (**E**), and the percentage of TUNEL-positive cells (**F**). Data are presented as mean ± SD (*n* = 3). Statistical significance is indicated in the graph. **** *p* < 0.0001 vs. the control group; ^##^ *p* < 0.01, ^###^ *p* < 0.001, and ^####^ *p* < 0.0001 vs. the model group.

Immunofluorescence staining was performed to examine p-STAT3 and p-EGFR expression in ulcer tissues (Figure 12A,B). In the control group, only weak p-STAT3 and p-EGFR signals were detected. RAU induction markedly increased p-STAT3-positive and p-EGFR-positive staining in the ulcer area, indicating activation of STAT3/EGFR-associated signaling after mucosal injury. EA treatment reduced p-STAT3 staining in a dose-related manner, with the most pronounced reduction observed in the EA-HD group (Figure 12A,D). A similar pattern was observed for p-EGFR. p-EGFR positivity was clearly lower in the EA-MD and EA-HD groups than in the model group, whereas the reduction was smaller in the EA-LD group (Figure 12B,E).

TUNEL staining was used to assess apoptosis in ulcer tissues (Figure 12C). The model group showed abundant TUNEL-positive cells, whereas only sparse signals were observed in the control group. EA treatment reduced TUNEL positivity, and the decrease was most evident in the EA-MD and EA-HD groups (Figure 12F). The CCDP group showed a comparable reduction in p-STAT3, p-EGFR, and TUNEL signals.

Together, these results indicate that EA attenuated injury-associated STAT3/EGFR activation and reduced apoptosis in RAU ulcer tissues.

### 3.16. EA Protects HOK Cells Against H_2_O_2_-Induced Oxidative Injury

**Figure 13:**
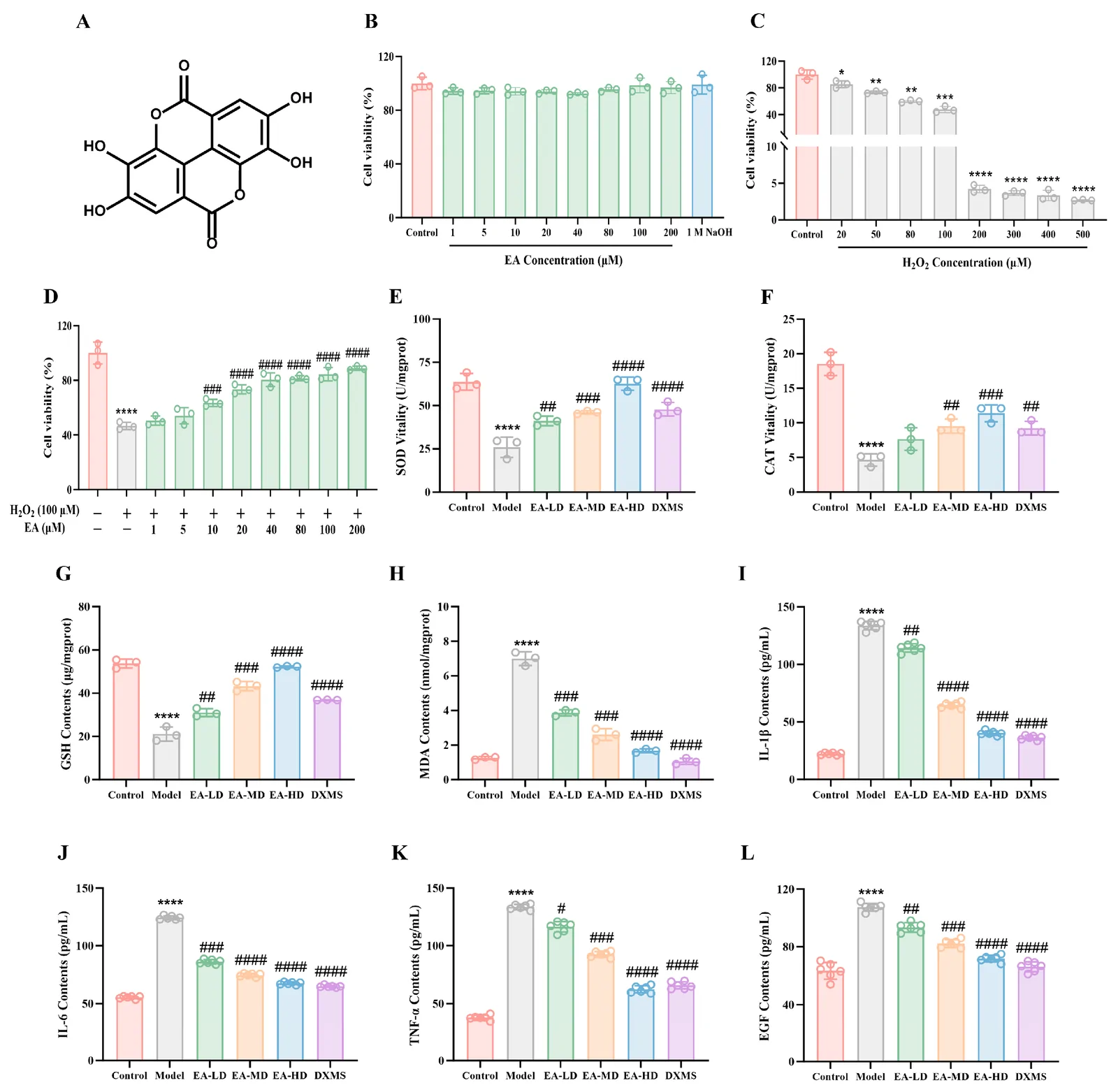
EA protects HOK cells against H_2_O_2_-induced oxidative injury by restoring redox homeostasis and attenuating inflammatory responses. (**A**) Chemical structure of EA. (**B**) Effects of different concentrations of EA and the corresponding NaOH vehicle on HOK cell viability. (**C**) Effects of different concentrations of H_2_O_2_ on HOK cell viability. (**D**) Protective effects of EA on cell viability in H_2_O_2_-injured HOK cells. (**E**–**H**) Effects of EA on intracellular redox-related indices, including SOD activity (**E**), CAT activity (**F**), GSH content (**G**), and MDA content (**H**). (**I**–**L**) Effects of EA on secreted inflammatory and repair-related mediators, including IL-1β (**I**), IL-6 (**J**), TNF-α (**K**), and EGF (**L**). Data are presented as mean ± SD (*n* = 3 or *n* = 6). * *p* < 0.05, ** *p* < 0.01, *** *p* < 0.001, and **** *p* < 0.0001 vs. the control group; ^#^ *p* < 0.05, ^##^ *p* < 0.01, ^###^ *p* < 0.001, and ^####^ *p* < 0.0001 vs. the model group.

HOK cells were used to evaluate whether EA protected oral epithelial cells from oxidative injury. The chemical structure of EA is shown in Figure 13A. EA cytocompatibility was first assessed over a concentration range of 1–200 μM. EA alone did not reduce HOK cell viability, and the corresponding NaOH vehicle showed no detectable cytotoxicity under the same conditions (Figure 13B). These results indicated that EA was well tolerated by HOK cells within the tested range.

HOK cells were then exposed to increasing concentrations of H_2_O_2_ to establish an oxidative injury model. H_2_O_2_ reduced cell viability in a concentration-dependent manner (Figure 13C). A concentration of 100 μM H_2_O_2_ produced a stable reduction in viability while retaining enough viable cells for intervention experiments. This concentration was therefore used for subsequent model establishment.

EA was next tested in H_2_O_2_-injured HOK cells. Cell viability was markedly lower in H_2_O_2_-exposed cells than in control cells. EA treatment restored cell viability in a concentration-dependent manner, with significant protection observed from 10 μM onward (Figure 13D). Based on these results, EA concentrations of 10, 20, and 80 μM were selected for the EA-LD, EA-MD, and EA-HD groups, respectively, in subsequent assays. These findings show that EA protected HOK cells against H_2_O_2_-induced oxidative injury.

### 3.17. EA Restores Redox Homeostasis and Attenuates Inflammatory Responses in H_2_O_2_-Injured HOK Cells

Intracellular redox indices were measured to determine whether EA reduced oxidative stress in H_2_O_2_-injured HOK cells (Figure 13E–H). Compared with control cells, H_2_O_2_-exposed cells showed lower SOD and CAT activities and GSH content, together with greater MDA accumulation. EA treatment partly reversed these changes. SOD and CAT activities and GSH content were increased in the EA-treated groups compared with the model group, whereas MDA accumulation was reduced. The strongest overall recovery was observed in the EA-HD group. The DXMS group showed a similar trend.

Inflammatory and repair-related mediators were then examined in the culture supernatant (Figure 13I,L). H_2_O_2_ exposure increased IL-1β, IL-6, TNF-α, and EGF levels, indicating that oxidative injury was accompanied by inflammatory activation and an altered repair-associated response. EA treatment reduced IL-1β, IL-6, and TNF-α levels, with stronger effects at higher concentrations. EGF was also reduced after EA treatment, particularly in the EA-MD and EA-HD groups. These results show that EA restored redox homeostasis and attenuated inflammatory activation in H_2_O_2_-injured HOK cells.

Collectively, these results demonstrate that EA alleviates H_2_O_2_-induced epithelial cell injury by restoring redox homeostasis and suppressing oxidative stress-associated inflammatory activation in HOK cells.

### 3.18. EA Alleviates Mitochondrial Dysfunction, ROS Accumulation, and Lipid Droplet Accumulation in H_2_O_2_-Injured HOK Cells

**Figure 14:**
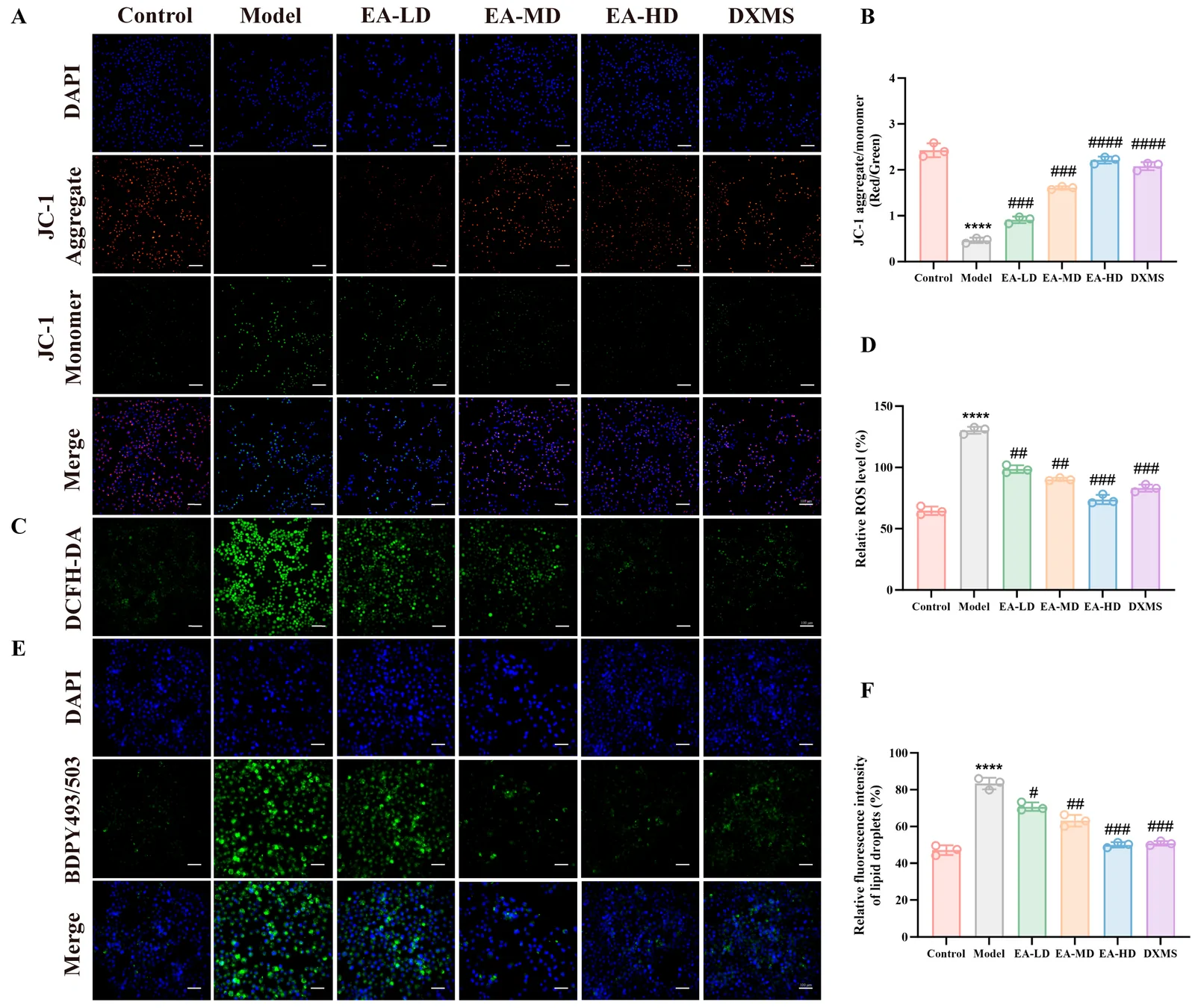
EA alleviates mitochondrial dysfunction, ROS accumulation, and lipid droplet accumulation in H_2_O_2_-injured HOK cells. (**A**) Representative JC-1 fluorescence images showing mitochondrial membrane potential in HOK cells. Red fluorescence indicates JC-1 aggregates, whereas green fluorescence indicates JC-1 monomers. (**B**) Quantification of the JC-1 aggregate/monomer fluorescence ratio. (**C**) Representative DCFH-DA fluorescence images showing intracellular DCF fluorescence. (**D**) Quantification of ROS fluorescence intensity. (**E**) Representative BODIPY 493/503 fluorescence images showing lipid droplet accumulation. (**F**) Quantification of BODIPY 493/503 fluorescence intensity. Data are presented as mean ± SD (*n* = 3). **** *p* < 0.0001 vs. the control group; ^#^ *p* < 0.05, ^##^ *p* < 0.01, ^###^ *p* < 0.001, and ^####^ *p* < 0.0001 vs. the model group.

Fluorescence staining was used to evaluate mitochondrial membrane potential, intracellular ROS, and lipid droplet accumulation in H_2_O_2_-injured HOK cells (Figure 14). JC-1 staining showed strong red aggregate fluorescence and weak green monomer fluorescence in the control group, indicating preserved mitochondrial membrane potential (Figure 14A). H_2_O_2_ exposure reduced red fluorescence, increased green fluorescence, and lowered the JC-1 aggregate/monomer ratio (Figure 14A,B). EA treatment restored the red/green fluorescence ratio, with the strongest recovery observed in the EA-HD group.

Intracellular ROS levels were assessed using DCFH-DA staining. Compared with control cells, H_2_O_2_-exposed cells showed markedly increased green fluorescence, indicating ROS accumulation (Figure 14C,D). EA treatment reduced ROS fluorescence intensity, and the reduction was most evident in the EA-HD group. The DXMS group showed a similar decrease in DCF fluorescence.

Lipid droplet accumulation was evaluated using BODIPY 493/503 staining. H_2_O_2_ exposure increased BODIPY-positive fluorescence, indicating lipid droplet accumulation in injured HOK cells (Figure 14E,F). EA treatment reduced lipid droplet fluorescence, with the strongest effect observed in the EA-HD group. A similar pattern was observed in the DXMS group.

Together, these fluorescence results show that EA preserved mitochondrial membrane potential, reduced ROS accumulation, and reduced lipid droplet accumulation in H_2_O_2_-injured HOK cells.

### 3.19. EA Promotes Epithelial Migration and Suppresses Apoptosis in H_2_O_2_-Injured HOK Cells

**Figure 15:**
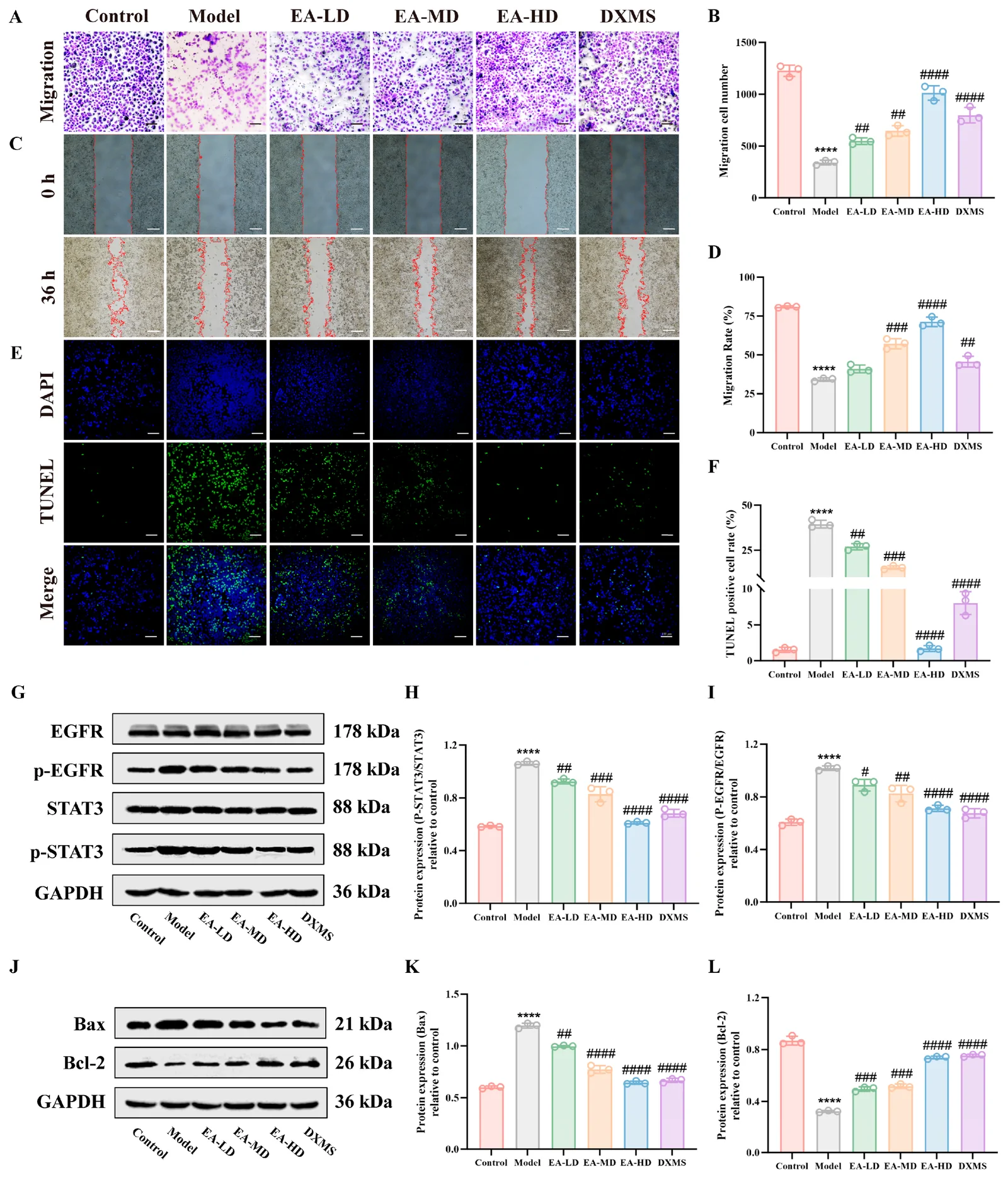
EA promotes epithelial migratory repair, suppresses apoptosis, and regulates STAT3/EGFR signaling in H_2_O_2_-injured HOK cells. (**A**) Representative images from the Transwell migration assay in HOK cells. (**B**) Quantification of migrated cells. (**C**) Representative images from the wound-healing assay at 0 h and 36 h. (**D**) Quantification of the wound closure rate. (**E**) Representative TUNEL staining images showing apoptotic HOK cells. Nuclei were counterstained with DAPI. (**F**) Quantification of the percentage of TUNEL-positive cells. (**G**) Representative Western blot bands of STAT3, p-STAT3, EGFR, p-EGFR, and GAPDH. (**H**,**I**) Quantification of relative STAT3 and EGFR phosphorylation levels, expressed as p-STAT3/STAT3 (**H**) and p-EGFR/EGFR (**I**). (**J**) Representative Western blot bands of Bax, Bcl-2, and GAPDH. (**K**,**L**) Quantification of apoptosis-related proteins, including Bax (**K**) and Bcl-2 (**L**). Data are presented as mean ± SD (*n* = 3). **** *p* < 0.0001 vs. the control group; ^#^ *p* < 0.05, ^##^ *p* < 0.01, ^###^ *p* < 0.001, and ^####^ *p* < 0.0001 vs. the model group.

Transwell and wound-healing assays were used to assess the effect of EA on epithelial migratory repair (Figure 15A–D). In the Transwell assay, many HOK cells migrated through the membrane in the control group, whereas H_2_O_2_ exposure markedly reduced the number of migrated cells (Figure 15A,B). EA treatment increased the number of migrated cells compared with the model group, with the strongest effect observed in the EA-HD group. The DXMS group showed a similar improvement.

In the wound-healing assay, scratch widths were comparable among groups at 0 h. After 36 h, the control group showed clear wound closure, whereas the model group retained a wide wound gap (Figure 15C,D). EA treatment increased the wound closure rate, particularly in the EA-MD and EA-HD groups. These results show that EA improved the migratory capacity of H_2_O_2_-injured HOK cells.

TUNEL staining was used to assess apoptosis (Figure 15E,F). The control group showed only sparse TUNEL-positive cells, whereas H_2_O_2_ exposure increased TUNEL positivity. EA treatment reduced the proportion of TUNEL-positive cells, with the most pronounced reduction observed in the EA-HD group. DXMS also reduced TUNEL positivity compared with the model group. These results indicate that EA enhanced epithelial migration and reduced apoptosis in H_2_O_2_-injured HOK cells.

### 3.20. EA Regulates STAT3/EGFR Signaling and Apoptosis-Related Proteins in H_2_O_2_-Injured HOK Cells

Western blot analysis was performed to examine STAT3/EGFR signaling in H_2_O_2_-injured HOK cells (Figure 15G–I). The p-STAT3/STAT3 and p-EGFR/EGFR ratios were higher in H_2_O_2_-exposed cells than in control cells, indicating activation of STAT3/EGFR-associated signaling. EA treatment reduced the phosphorylation levels of both STAT3 and EGFR, with the strongest reduction observed in the EA-HD group. This pattern was consistent with the in vivo immunofluorescence results.

Apoptosis-related proteins were then examined (Figure 15J–L). H_2_O_2_ exposure upregulated Bax and downregulated Bcl-2 compared with the control group. EA treatment decreased Bax expression and restored Bcl-2 expression, with the most pronounced effect observed in the EA-HD group. DXMS showed a comparable trend.

Together, the cell-level results show that EA improved epithelial migration, reduced apoptosis, and attenuated STAT3/EGFR activation in H_2_O_2_-injured HOK cells. These findings are consistent with the protective effects observed in EA-treated RAU rats.

## 4. Discussion

Oral mucosal ulcer repair requires coordinated resolution of oxidative stress and inflammation, repair of epithelial injury, and remodeling of the extracellular matrix. In RAU-relevant mucosal injury, excessive ROS generation and inflammatory activation may disrupt this transition and delay epithelial restitution. These processes provide a rationale for investigating interventions that support both redox balance and epithelial repair [42,43,44]. In the present study, EA was selected from the serum-exposed constituent profile of YMKY and evaluated as the principal intervention compound. EA accelerated ulcer closure in an acetic acid-induced oral ulcer model, improved epithelial integrity and collagen organization, and protected HOK cells against H_2_O_2_-induced oxidative injury. Previous research has investigated EA in a rat model of chemotherapy-associated oral mucositis [17]. The present study extends this work by combining repair outcomes in a chemical injury model with cellular assessments of oxidative injury and STAT3/EGFR-associated signaling. These findings support the repair-promoting activity of EA under the experimental conditions examined, without establishing its efficacy against the recurrent disease process of human RAU.

Serum pharmacochemistry identified EA as an absorbed prototype constituent of YMKY. This finding supported its selection for further investigation, but does not establish that EA accounts for the overall therapeutic activity of the multicomponent formulation. Molecular docking prioritized STAT3 and EGFR as putative EA-interacting targets, whereas MD simulation and MM/GBSA analysis evaluated the stability and energetic favorability of the predicted complexes. DFT analysis provided complementary electronic-structure information that helped rationalize the polar interaction features of EA in the docking models, rather than independent evidence of direct STAT3/EGFR target engagement. Collectively, these computational findings generate hypotheses for experimental testing, but do not establish target-specific recognition or direct binding.

A consistent finding of this study was the improvement in redox-related indices alongside reduced inflammatory mediator levels. RAU has been associated with increased oxidative burden, impaired antioxidant defense, and cytokine dysregulation involving TNF-α and IL-6 [5,40,45]. ROS can function as physiological signaling molecules, but excessive ROS production promotes lipid peroxidation, mitochondrial dysfunction, apoptosis, and inflammatory amplification [46]. In rats, EA increased serum SOD and CAT activities and GSH levels while reducing MDA levels. Similar changes were observed in H_2_O_2_-injured HOK cells, together with reduced DCF fluorescence and preservation of mitochondrial membrane potential. These changes, accompanied by reduced inflammatory mediator levels, are consistent with a cellular environment more favorable for epithelial survival and repair. This interpretation is consistent with previous reports that EA and its metabolites regulate oxidative stress, inflammation, apoptosis, and metabolic homeostasis [15,47]. The mitochondrial findings are also biologically plausible because ellagic-acid-related metabolites, such as urolithin A, have been linked to mitochondrial quality control [48]. However, the serum measurements do not directly establish antioxidant effects within ulcer tissues, and the cellular endpoints cannot determine whether mitochondrial preservation precedes or follows the reduction in oxidative stress. Furthermore, decreased BODIPY 493/503 fluorescence indicates reduced accumulation of neutral lipids/lipid droplets but does not demonstrate restoration of lipid metabolic flux.

EA also appeared to support epithelial repair processes. In vivo, EA improved epithelial continuity and collagen deposition, while in vitro it improved outcomes in migration assays and reduced apoptosis. These effects are important because re-epithelialization requires both inflammatory resolution and the preservation of viable, migratory epithelial cells. EGFR-mediated epithelial movement and STAT3-dependent keratinocyte repair programs support the relevance of examining these pathways in mucosal healing [9,12,13]. However, the observed reduction in p-STAT3 and p-EGFR should not be interpreted as evidence that pathway inhibition directly promotes repair. One possible explanation is that EA attenuates injury-associated signaling as oxidative and inflammatory stress decreases. Alternatively, the phosphorylation changes may be secondary to improved tissue condition rather than the primary drivers of repair [49]. The present data cannot distinguish these possibilities or establish the temporal relationship between signaling changes and wound closure. Thus, normalization of injury-associated signaling remains a working interpretation rather than a demonstrated mechanism.

Several limitations should be acknowledged. Although the acetic acid-induced oral ulcer model reproduces acute oral mucosal injury and repair-related features relevant to RAU, it does not capture the spontaneous recurrence, chronic immune predisposition, or patient-specific triggers of human RAU. The persistence of ulceration in the model group over the seven-day observation period may reflect the severity of the initial chemical injury, and the complete spontaneous healing course was not established within this period. Accordingly, the reductions in ulcer area and improvements in tissue repair observed with EA support its repair-promoting activity in this experimental injury model, while its applicability to human RAU requires further investigation. The H_2_O_2_-induced HOK injury model likewise isolates oxidative epithelial injury without reproducing the multicellular environment of an oral ulcer. Direct binding of EA to STAT3 or EGFR requires confirmation through biochemical binding or cellular target-engagement assays. Separately, pathway perturbation and rescue experiments are needed to determine whether STAT3/EGFR signaling is functionally required for the protective effects of EA. In addition, EA has limited aqueous solubility, and its oral mucosal exposure after gavage remains unclear. Pharmacokinetic and tissue-distribution studies would strengthen the exposure-response interpretation. Finally, RAU recurrence may involve salivary microbiota disturbance, metabolic remodeling, and epithelial inflammatory memory, which were not addressed here [50,51]. Future studies should also evaluate whether local delivery improves mucosal exposure and whether the observed benefits extend to models more representative of recurrent oral ulceration.

Overall, this study supports EA as a candidate for promoting repair following experimental oral mucosal injury. Its protective effects were accompanied by improved redox-related indices, reduced inflammatory responses and apoptosis, and altered STAT3/EGFR phosphorylation. Establishing the causal contribution of these signaling changes remains an important next step.

## 5. Conclusions

**Figure 16:**
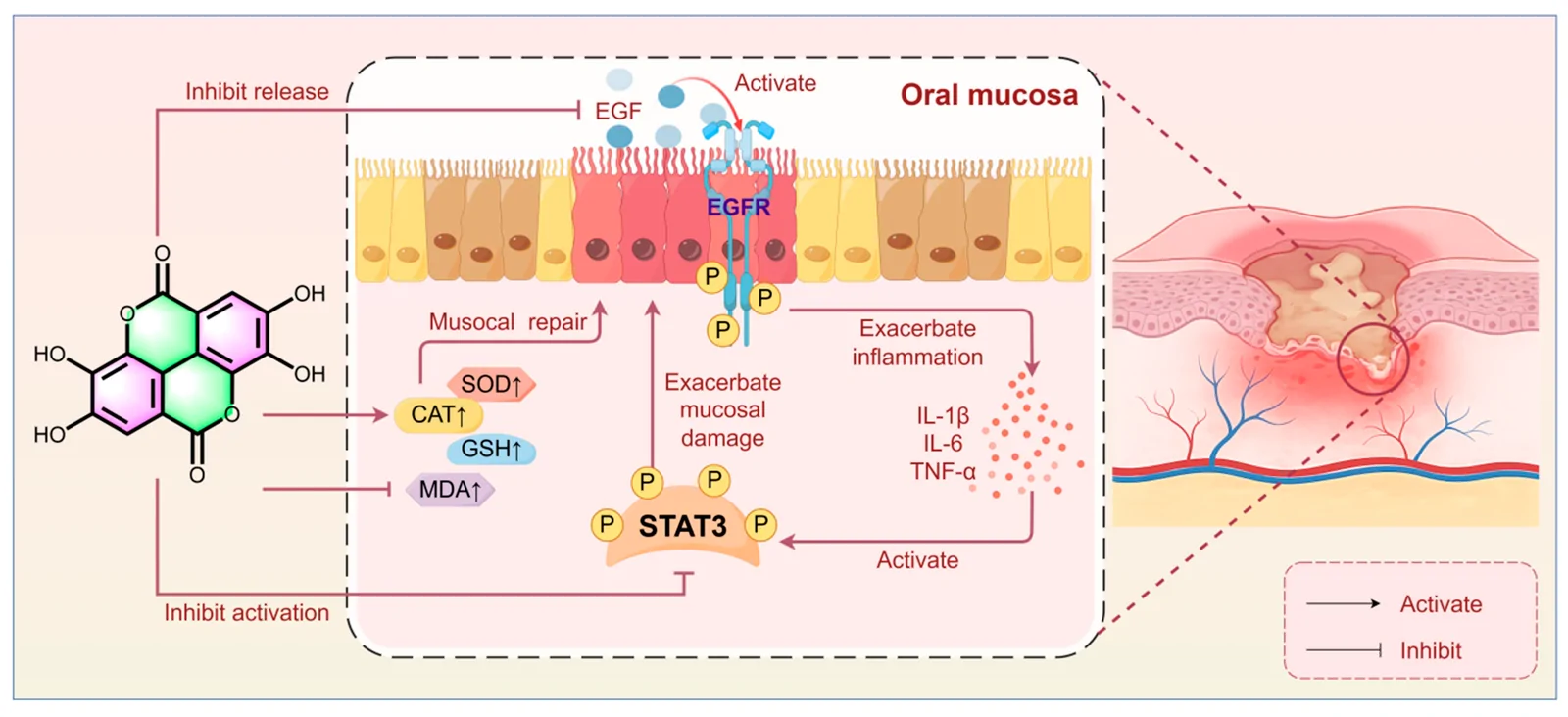
Proposed working model by which EA promotes healing of recurrent aphthous ulcers. EA promoted repair of acetic acid-induced oral ulcers in rats and protected H_2_O_2_-injured oral keratinocytes. These effects were accompanied by improved antioxidant indices (SOD, CAT, and GSH), reduced MDA and inflammatory cytokine levels (IL-1β, IL-6, and TNF-α), decreased apoptosis, and reduced STAT3 and EGFR phosphorylation.

In conclusion, this study identifies EA as a serum-exposed constituent of YMKY with repair-promoting potential in experimental oral mucosal injury. EA accelerated ulcer closure, improved epithelial integrity and collagen organization, improved systemic redox-inflammatory indices, and reduced apoptosis in rats with acetic acid-induced oral ulcers. Consistent with these in vivo findings, EA protected H_2_O_2_-injured HOK cells against oxidative and inflammatory injury, preserved mitochondrial membrane potential, improved migration-assay outcomes, and shifted apoptosis-related protein expression toward a pro-survival profile. The integrated evidence suggests that EA promotes experimental oral mucosal repair in association with improved redox-inflammatory indices and altered STAT3/EGFR phosphorylation; however, direct target engagement and the causal contribution of these signaling changes remain unconfirmed. The findings support further investigation of EA for oral mucosal repair but do not establish efficacy against human RAU (Figure 16).

## Figures and Tables

**Table 1:** Serum-exposed prototype constituents used for EA source tracing, identified by UPLC-Q Exactive-Orbitrap HRMS.

Peak No.	t_R_ (min)	Compound	Chemical Formula	Ion Type	Theoretical Value (*m*/*z*)	Experimental Value (*m*/*z*)	Error/×10^−6^	Major Fragment Ions (*m*/*z*)	Category	Source	Refs.
7	2.48	Pantothenic acid	C_9_H_17_NO_5_	[M + H]^+^	220.1179	220.1181	0.91	220.1181; 202.1076; 184.0968; 116.0347; 90.0555	Organic acids	*P. emblica*	[31]
9	3.73	Pyrogallol *	C_6_H_6_O_3_	[M + H]^+^	127.0390	127.0394	3.15	127.0393; 109.0288; 99.0445; 81.0342; 53.0394	Organic acids	*P. emblica*	[32]
22	17.08	Rehmapicroside	C_16_H_26_O_8_	[M - H]^−^	345.1544	345.1559	4.35	345.1554; 301.1655; 183.1017; 161.0449; 113.0238	Terpenoids	*R. glutinosa*	[33]
36	17.68	Liquiritigenin *	C_15_H_12_O_4_	[M + H]^+^	257.0808	257.0811	1.17	257.0809; 147.0443; 137.0235; 119.0496	Flavonoids	*G. uralensis*	[34]
37	17.77	Ellagic acid *	C_14_H_6_O_8_	[M - H]^−^	300.9978	300.9990	3.99	300.9990; 257.0091; 229.0140; 185.0240	Phenolic acids	*P. emblica*, *P. lactiflora*	[35,36]
38	17.91	Liquiritigenin-7-*O*-β-D-apiofuranosyl-4′-*O*-β-D-glucopyranoside	C_26_H_30_O_13_	[M - H]^−^	549.1603	549.1617	2.55	549.1613; 255.0662; 153.0186; 135.0079; 119.0494	Flavonoids	*G. uralensis*	[37]
41	18.36	Salicylic acid	C_7_H_6_O_3_	[M - H]^−^	137.0233	137.0236	2.19	137.0236; 93.0336; 65.0386	Organic acids	*O. japonicus*	[38]
49	20.27	Kaempferol-7-*O*-β-D-glucopyranoside *	C_21_H_20_O_11_	[M - H]^−^	447.0922	447.0938	3.58	447.0936; 285.0408; 257.0447; 169.0132; 151.0030	Flavonoids	*P. emblica*	[14]
55	20.90	Liquiritin *	C_21_H_22_O_9_	[M - H]^−^	417.1180	417.1197	4.08	417.1192; 255.0663; 153.0186; 135.0079; 119.0494	Flavonoids	*G. uralensis*	[34]
72	35.19	(15*Z*)-9,12,13-Trihydroxy-15-octadecenoic acid	C_18_H_34_O_5_	[M - H]^−^	329.2323	329.2334	3.34	329.2334; 211.1335; 171.1020; 139.1120; 99.0806	Organic acids	*G. uralensis*	[34]
80	43.34	Kanzonol K	C_26_H_28_O_6_	[M + H]^+^	437.1959	437.1941	−4.12	437.1938; 303.1194	Flavonoids	*G. uralensis*	[39]
82	49.18	Oleamide	C_18_H_35_NO	[M + H]^+^	282.2791	282.2794	1.06	282.2792; 265.2526; 247.2420; 135.1174; 95.0859	Fatty amides	*G. uralensis*	[34]
83	49.47	Hexadecanamide	C_16_H_33_NO	[M + H]^+^	256.2635	256.2637	0.78	256.2640; 102.0917; 88.0762; 57.0707	Fatty amides	*G. uralensis*	[34]
84	55.88	Erucamide	C22H43NO	[M + H]^+^	338.3417	338.3420	0.89	338.3416; 321.3150; 303.3050; 97.1017; 69.0706	Fatty amides	*G. uralensis*	[34]

Note: * indicates compounds confirmed by comparison with reference standards.

## Data Availability

Data are available from the authors.

## Supplementary Materials

The following supporting information can be downloaded at: https://www.mdpi.com/article/10.3390/antiox15101275/s1, Figure S1: UPLC-Q Exactive-Orbitrap HRMS total ion chromatograms of YMKY extract and blank serum in positive and negative ion modes; Figure S2: Chemical structures of the compounds identified in YMKY by UPLC-Q Exactive-Orbitrap HRMS; Figure S3: Term-gene association networks (cnetplots) for enriched GO terms and KEGG pathways; Figure S4: Similarity networks (emapplots) of enriched GO terms and KEGG pathways; Figure S5: MAPK signaling pathway; Figure S6: HIF-1 signaling pathway; Figure S7: Adherens junction pathway; Figure S8: JAK-STAT signaling pathway; Figure S9: Inflammatory mediator regulation of TRP channels pathway; Figure S10: Effect of EA on body weight in rats with RAU; Figure S11: Effect of EA on food intake in rats with RAU; Table S1: Compounds in YMKY identified by UPLC-Q Exactive-Orbitrap HRMS [14,31,32,33,34,35,36,37,38,39,52,53,54,55,56,57,58,59,60,61,62,63,64,65,66,67,68,69,70,71,72,73,74,75,76,77,78,79,80,81,82,83,84]; Table S2: Molecular docking binding energies and ligand efficiency of serum-absorbed prototype constituents against STAT3 and EGFR; Table S3: Predicted drug-likeness and physicochemical properties of the serum-absorbed prototype constituents; Table S4: In silico ADMET profiles of the serum-absorbed prototype constituents.

## Abbreviations

The following abbreviations are used in this manuscript:

RAU	Recurrent aphthous ulcers
EA	Ellagic acid
YMKY	Yumai kouyan heji
TNF-α	Tumor necrosis factor-alpha
IL	Interleukins
STAT3	Signal transducer and activator of transcription 3
EGFR	Epidermal growth factor receptor
TCM	Traditional chinese medicine
ESI	Electrospray ionization
ADMET	Absorption, distribution, metabolism, excretion, and toxicity
PPI	Protein–protein interaction
GO	Gene ontology
KEGG	Kyoto encyclopedia of genes and genomes
MD	Molecular dynamics
PDB	Protein data bank
DFT	Density functional theory
DOS	Density of states
HOMO	Highest occupied molecular orbital
LUMO	Lowest unoccupied molecular orbital
MEP	Molecular electrostatic potential
CCDP	Compound chlorhexidine-dexamethasone patch
EA-LD	EA-low dose
EA-MD	EA-medium dose
EA-HD	EA-high dose
HOK	Human oral keratinocytes
DXMS	Dexamethasone solution
MDA	Malondialdehyde
PBS	Phosphate-buffered saline
BCA	Bicinchoninic acid
SOD	Superoxide dismutase
CAT	Catalase
GSH	Glutathione
ELISA	Enzyme-linked immunosorbent assay
EGF	Epidermal growth factor
H&E	Hematoxylin and eosin
p-STAT3	Phosphorylated stat3
p-EGFR	Phosphorylated egfr
TUNEL	Terminal deoxynucleotidyl transferase-mediated dutp nick end labeling
MMP	Mitochondrial membrane potential
ROS	Reactive oxygen species
