# Supplementary material for: Ellagic Acid Promotes Repair of Acetic Acid-Induced Oral Ulcers in Rats: Associations with Redox-Inflammatory Responses and STAT3/EGFR Signaling
Source: Antioxidants (Basel). 2026 Oct 2;15(10):1275. doi: 10.3390/antiox15101275 (PMC13700818; doi:10.3390/antiox15101275)
Supplement: Supplementary file 1 [file antioxidants-15-01275-s001.zip › antioxidants-4533112-supplementary.pdf]

Ellagic Acid Promotes Repair of Acetic Acid-Induced Oral Ulcers in Rats: Associations with Redox-Inflammatory Responses and STAT3/EGFR Signaling

Jianfa Wu [1,2,†], Ying Cui [1,2,†], Huanang Chen [3], Shaofeng Shuai [1,2], Zhimin Zhou [1,2], Sijing Su [4], Yusen Hou [1,*], Ce Tang [1,2,4], Yi Zhang [1,2,4,*]

[1] State Key Laboratory of Southwestern Chinese Medicine Resources, School of Pharmacy, Chengdu University of Traditional Chinese Medicine, Chengdu 611137, China;

[2] Lab for Innovation & Effective Uses of Chinese Drug Germplasm Resources, School of Pharmacy, Chengdu University of Traditional Chinese Medicine, Chengdu 611137, China;

[3] School of Modern Chinese Medicine Industry, Chengdu University of Traditional Chinese Medicine, Chengdu 611137, China;

[4] School of Ethnic Medicine, Chengdu University of Traditional Chinese Medicine, Chengdu 611137, China;

[*]**Corresponding author:** houyusen@cdutcm.edu.cn (Y.H.); zhangyi@cdutcm.edu.cn (Y.Z.)

[†] These authors contributed equally to this work.

Contents

List of Figures

Figure S1. UPLC-Q Exactive-Orbitrap HRMS total ion chromatograms of YMKY extract and blank serum in positive and negative ion modes. 3

Figure S2. Chemical structures of the compounds identified in YMKY by UPLC-Q Exactive-Orbitrap HRMS. 4

Figure S3. Term-gene association networks (cnetplots) for enriched GO terms and KEGG pathways. 5

Figure S4. Similarity networks (emapplots) of enriched GO terms and KEGG pathways. 6

Figure S5. MAPK signaling pathway. 7

Figure S6. HIF-1 signaling pathway. 8

Figure S7. Adherens junction pathway. 9

Figure S8. JAK-STAT signaling pathway. 10

Figure S9. Inflammatory mediator regulation of TRP channels pathway. 11

Figure S10. Effect of EA on body weight in rats with RAU. 12

Figure S11. Effect of EA on food intake in rats with RAU. 13

List of Tables

Table S1. Compounds in YMKY identified by UPLC-Q Exactive-Orbitrap HRMS. 14

Table S2. Molecular docking binding energies and ligand efficiency of serum-absorbed prototype constituents against STAT3 and EGFR. 20

Table S3. Predicted drug-likeness and physicochemical properties of the serum-absorbed prototype constituents. 21

Table S4. In silico ADMET profiles of the serum-absorbed prototype constituents. 22

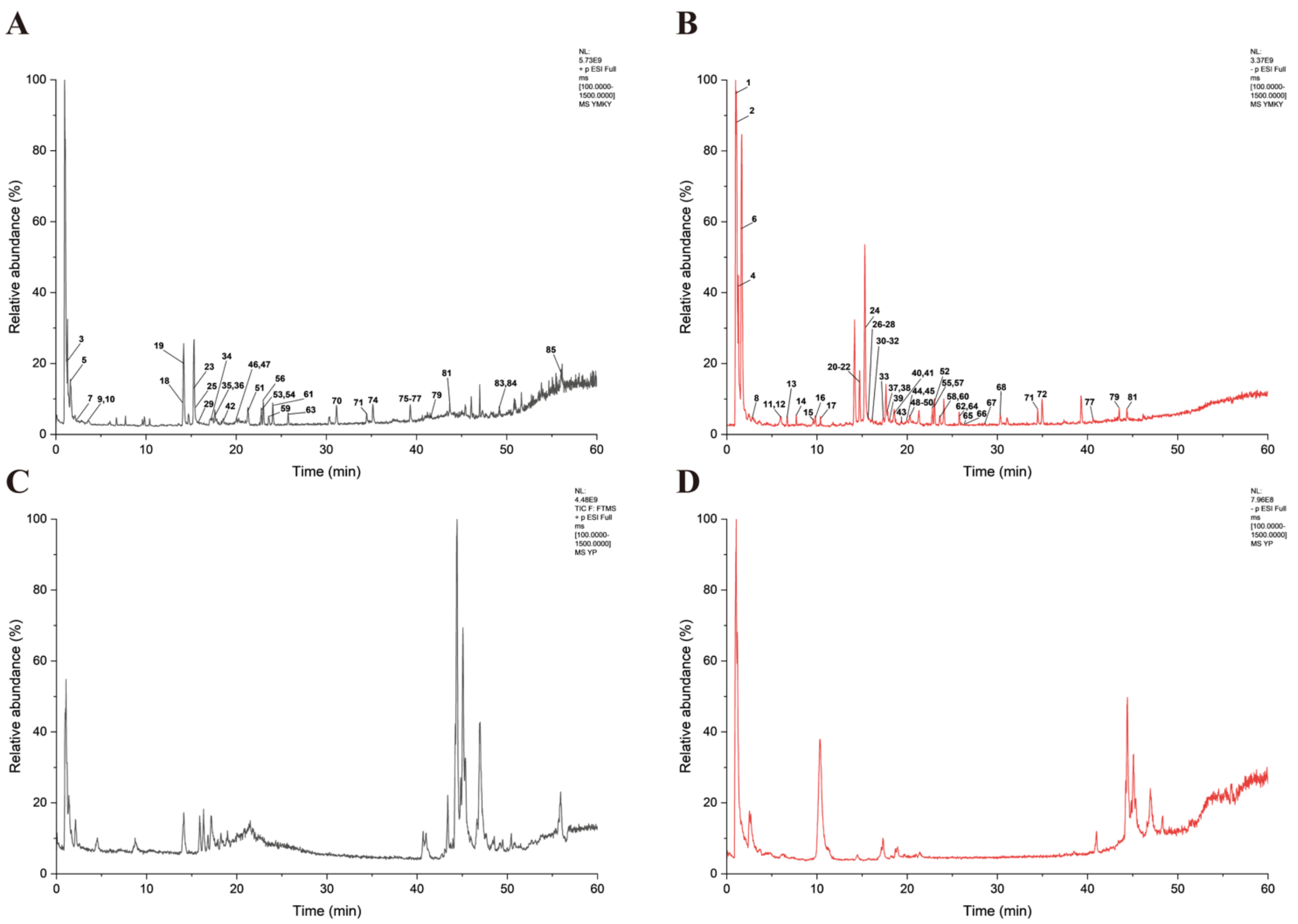

Figure S1. UPLC-Q Exactive-Orbitrap HRMS total ion chromatograms of YMKY extract and blank serum in positive and negative ion modes. (A) TIC of YMKY in the positive ESI mode. (B) TIC of YMKY in the negative ESI mode. (C) TIC of blank serum in the positive ESI mode. (D) TIC of blank serum in the negative ESI mode.

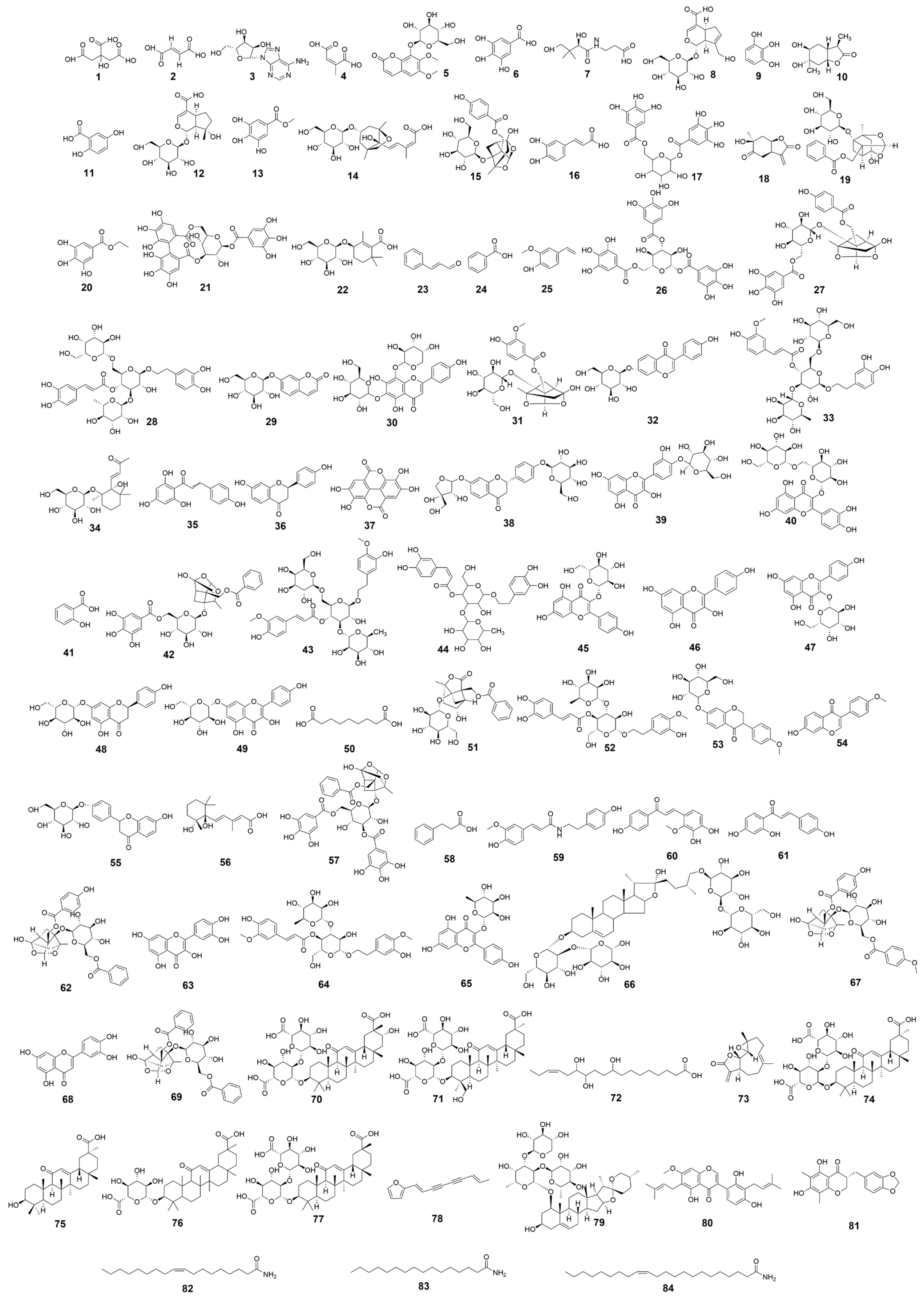

Figure S2. Chemical structures of the compounds identified in YMKY by UPLC-Q Exactive-Orbitrap HRMS.

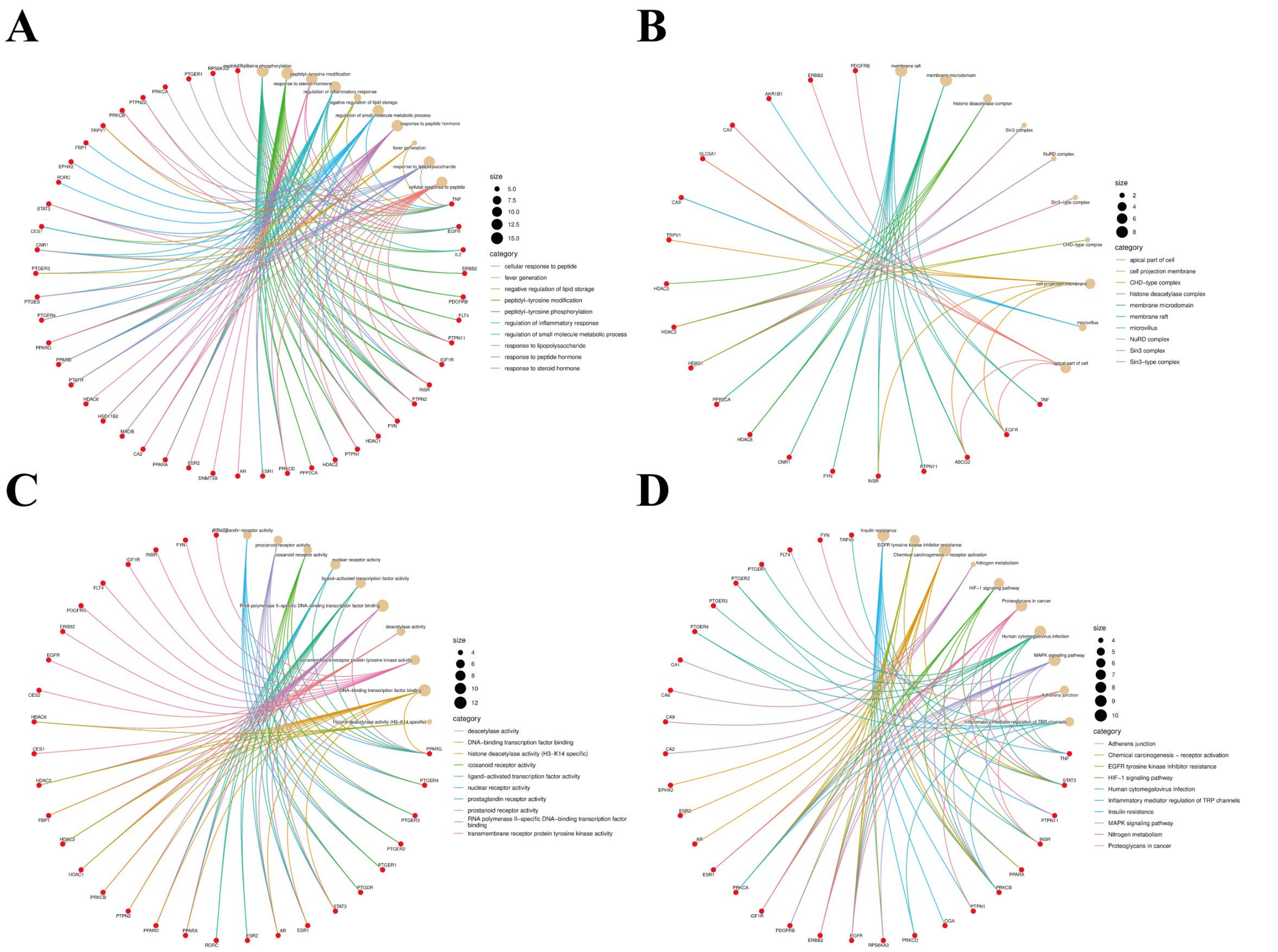

Figure S3. Term-gene association networks (cnetplots) for enriched GO terms and KEGG pathways. (A) GO BP cnetplot. (B) GO CC cnetplot. (C) GO MF cnetplot. (D) KEGG pathway cnetplot.

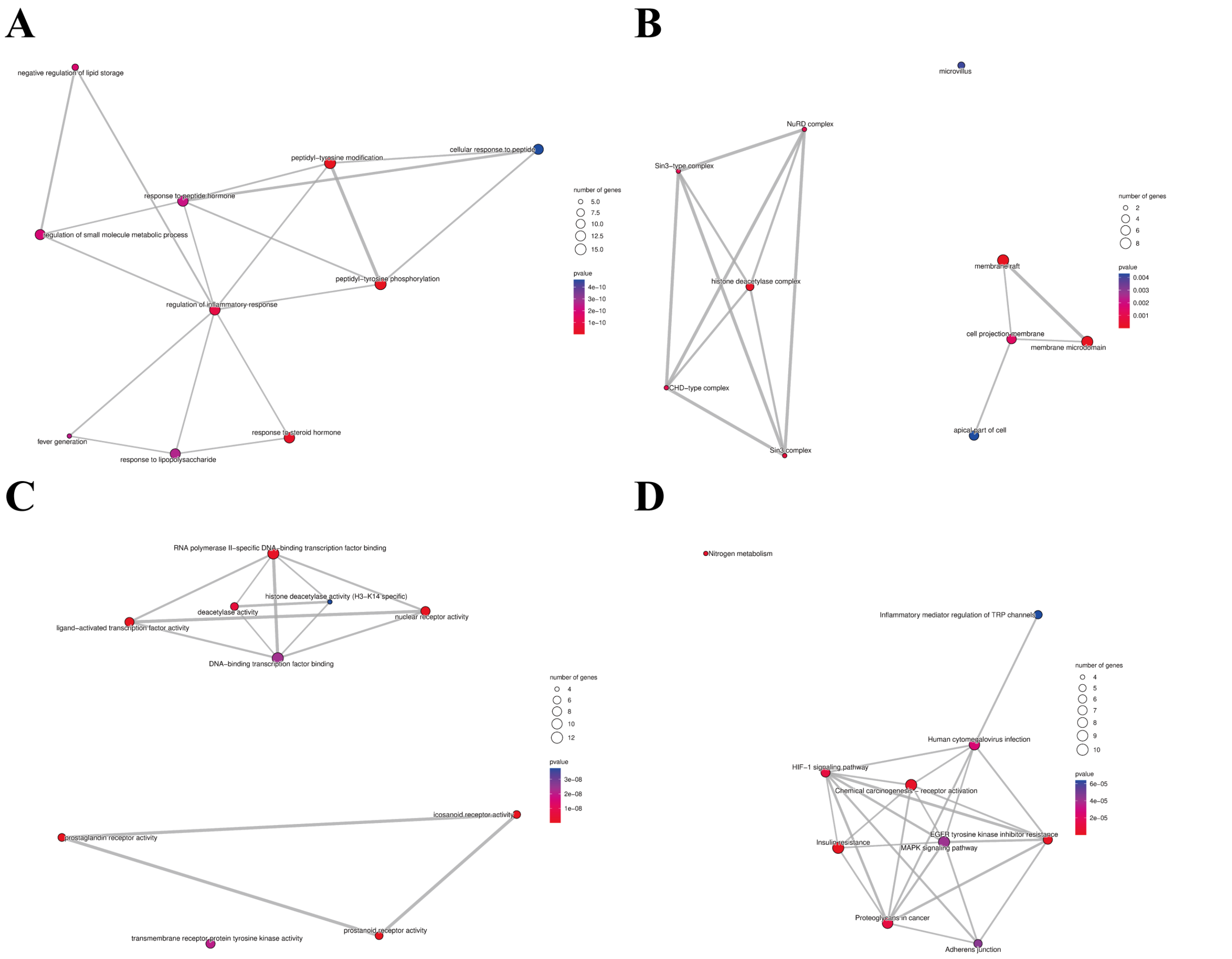

Figure S4. Similarity networks (emapplots) of enriched GO terms and KEGG pathways. (A) GO BP emapplot. (B) GO CC emapplot. (C) GO MF emapplot. (D) KEGG pathway emapplot.

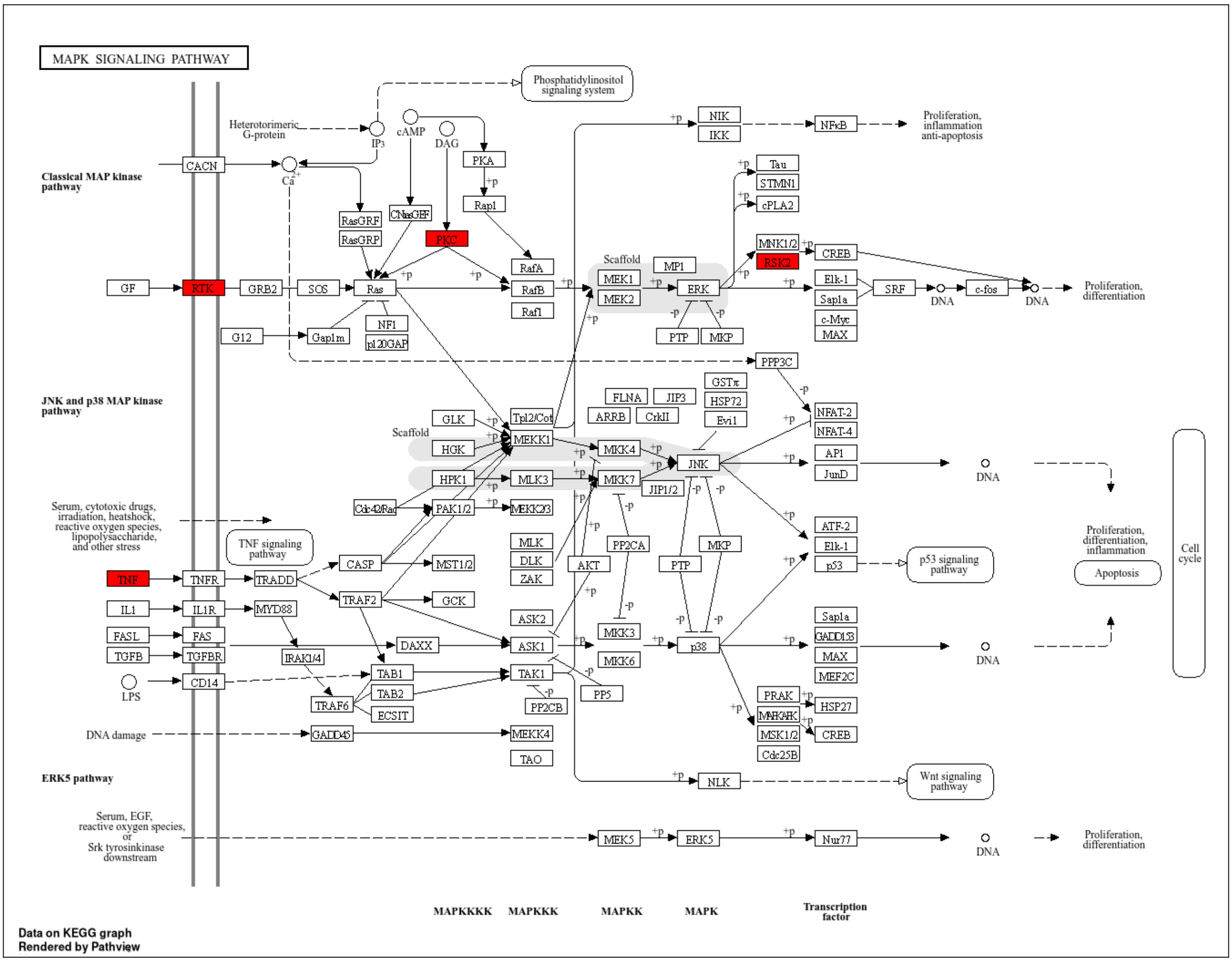

Figure S5. MAPK signaling pathway.

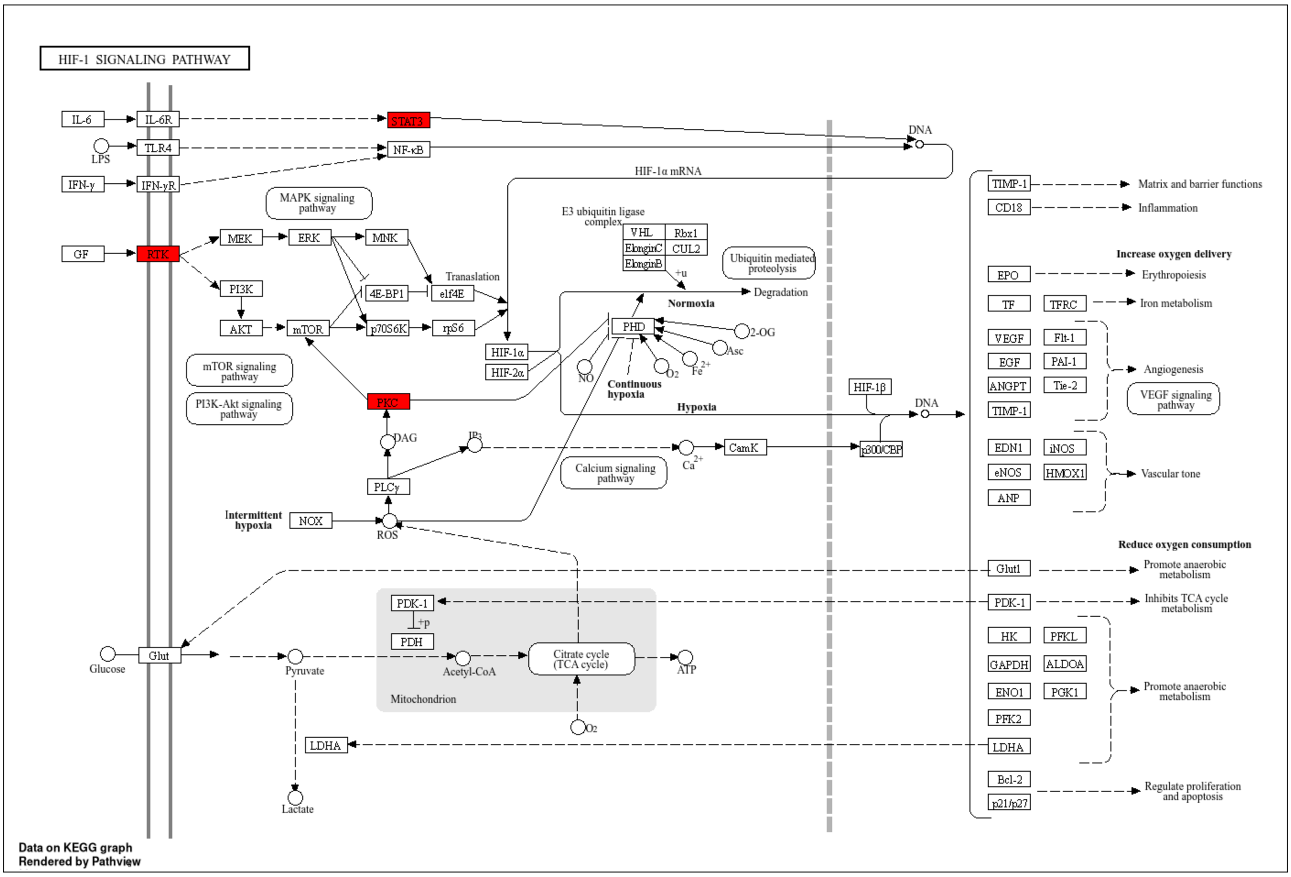

Figure S6. HIF-1 signaling pathway.

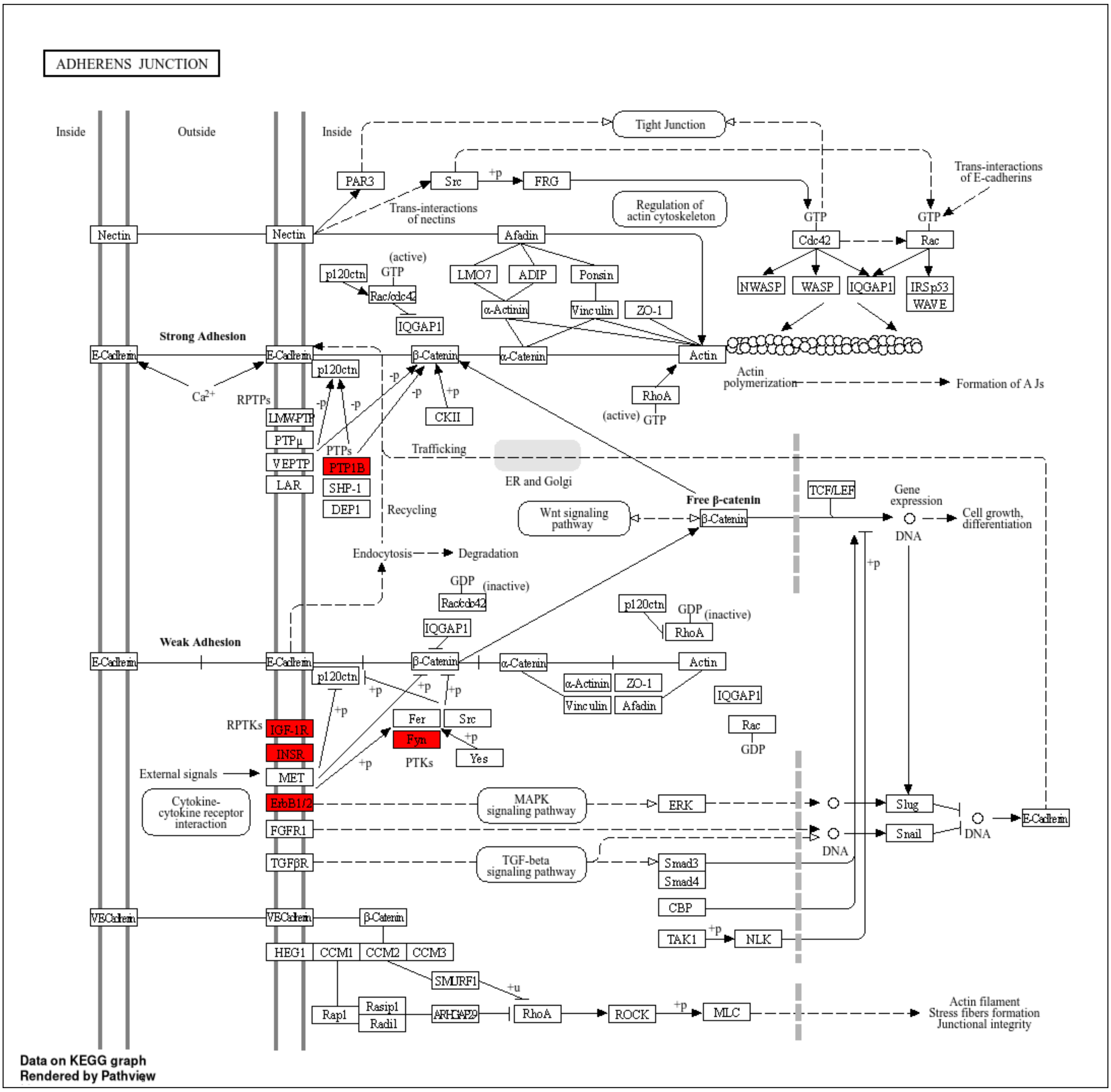

Figure S7. Adherens junction pathway.

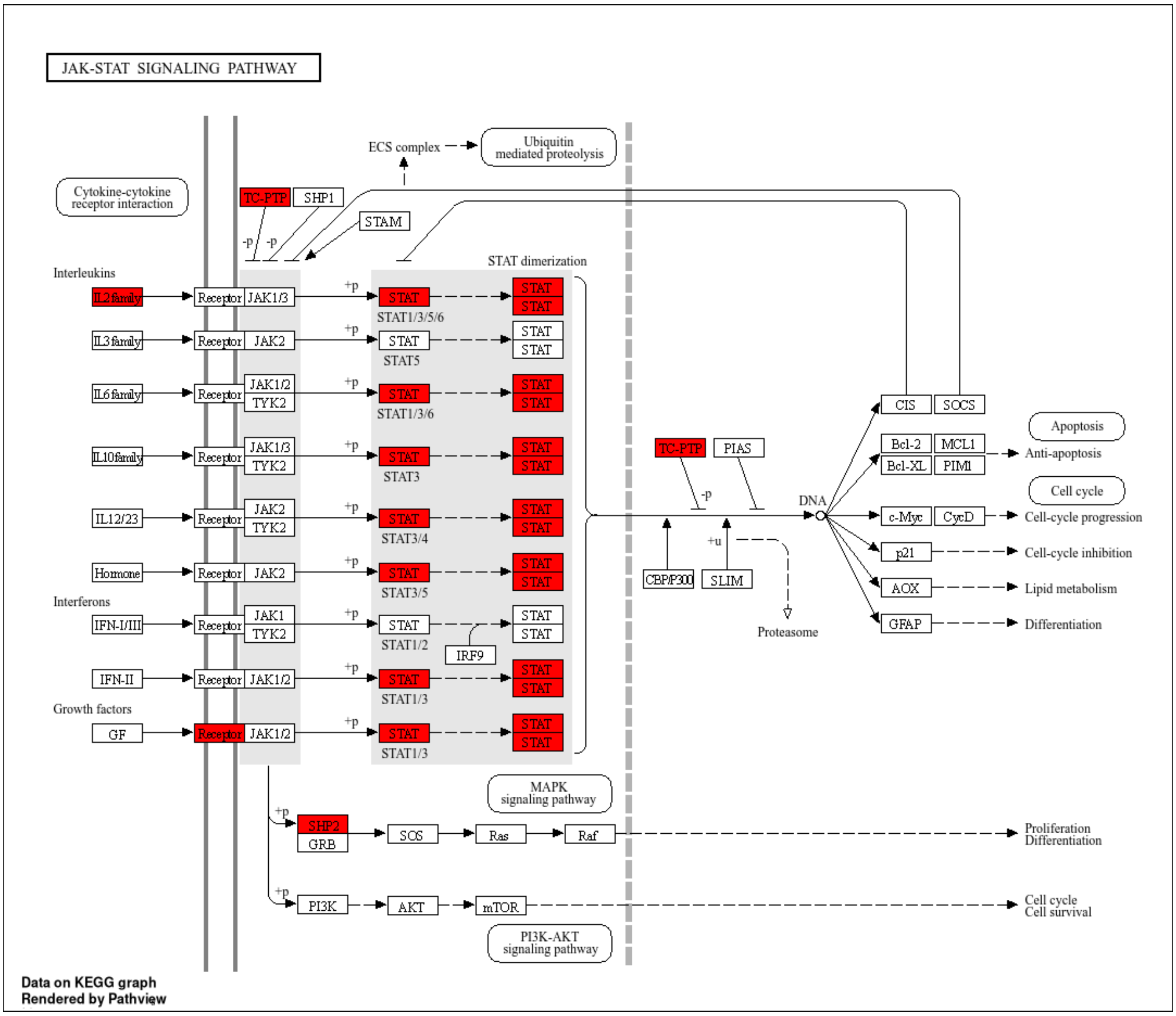

Figure S8. JAK-STAT signaling pathway.

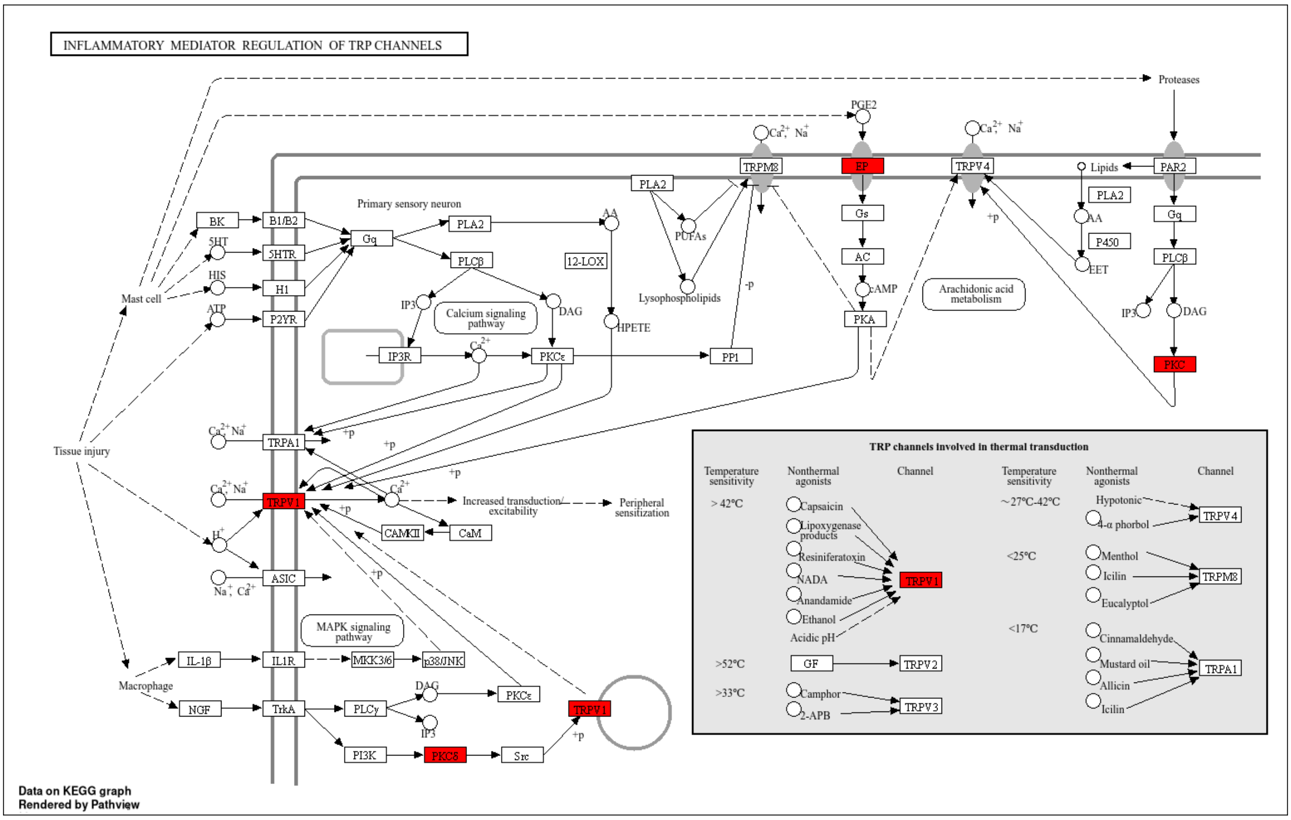

Figure S9. Inflammatory mediator regulation of TRP channels pathway.

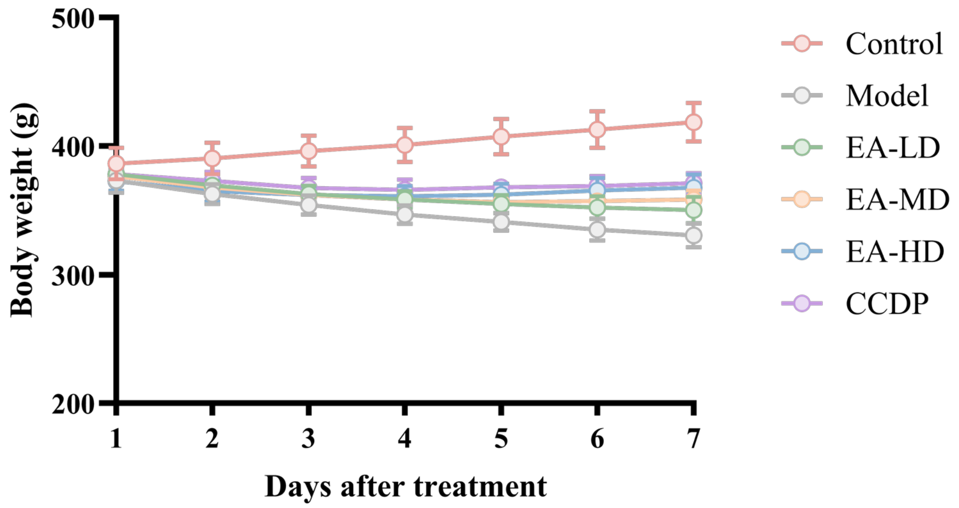

Figure S10. Effect of EA on body weight in rats with RAU.

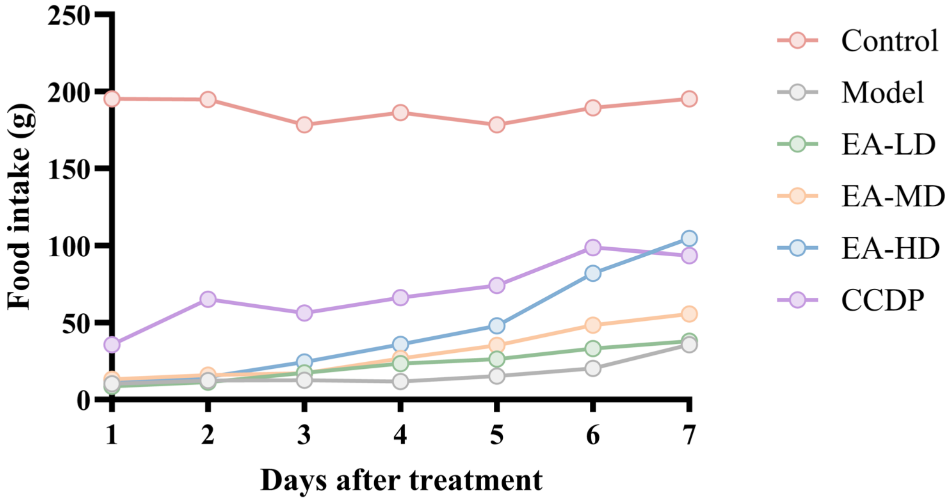

Figure S11. Effect of EA on food intake in rats with RAU.

Table S1. Compounds in YMKY identified by UPLC-Q Exactive-Orbitrap HRMS.

Peak No.	R_t (min)	Compound	Formula	Ion type	Theoretical value (m/z)	Experimental value (m/z)	Error /×10^{-6}	Major fragment ion (m/z)	Category	Source	Ref.
1	0.99	Citric acid	$C_6H_8O_7$	$[M-H]^-$	191.0186	191.0193	3.66	191.0193；147.0291；129.0188；85.0285；50.0128	Organic acid	R. *glutinosa*, *P. lactiflora*	[52,53]
2	1.04	Fumaric acid	$C_4H_4O_4$	$[M-H]^-$	115.0026	115.0029	2.61	115.0029；107.6126；71.0128	Organic acid	*P. lactiflora*	[52]
3	1.28	Adenosine	$C_{10}H_{13}N_5O_4$	$[M+H]^+$	268.1040	268.1040	0.00	268.1040；136.0620；119.0357	Nucleoside	*R. glutinosa*	[38]
4	1.51	Citraconic acid	$C_5H_6O_4$	$[M-H]^-$	129.0182	129.0186	3.10	129.0186；85.0285；71.0128	Organic acid	*P. emblica*	[54]
5	1.60	Fraxidinglucoside	$C_{17}H_{20}O_{10}$	$[M+H]^+$	385.1129	385.1113	-4.15	385.1102；223.0576；205.0471；125.0724；100.7243	Phenylpropanoids	*P. lactiflora*	[52]
6	1.64	Gallic acid*	$C_7H_6O_5$	$[M-H]^-$	169.0131	169.0134	1.78	169.0136；125.0236；107.0128；69.0335；67.0179	Organic acid	*P. emblica*	[54,55]
7	2.46	Pantothenic acid	$C_9H_{17}NO_5$	$[M+H]^+$	220.1179	220.1181	0.91	220.1181；202.1076；184.0968；116.0347；90.0555	Organic acid	*P. emblica*	[56]
8	2.88	Geniposidic acid	$C_{16}H_{22}O_{10}$	$[M-H]^-$	373.1129	373.1147	4.82	373.1147；211.0611；167.0709；149.0601；123.0443；71.0129；59.0128	Nitrogen-containing compound	*R. glutinosa*	[36]
9	3.58	Pyrogallol*	$C_6H_6O_3$	$[M+H]^+$	127.0390	127.0394	3.15	127.0393；109.0288；99.0445；81.0342；53.0394	Organic acid	*P. emblica*	[55]
10	3.72	Lactinolide	$C_{10}H_{16}O_4$	$[M+H]^+$	201.1121	201.1125	1.99	183.1019；137.0963；119.0859；109.1017	Terpenoids	*P. lactiflora*	[39]
11	5.91	Gentisic acid	$C_7H_6O_4$	$[M-H]^-$	153.0182	153.0188	3.92	153.0188；109.0286；108.0208；95.0129；85.0283	Organic acid	*R. glutinosa*	[57]
12	5.99	Mussaenosidic acid	$C_{16}H_{24}O_{10}$	$[M-H]^-$	375.1286	375.1300	3.73	375.1295；213.0765；169.0864；151.0756；59.0218	Terpenoids	*R. glutinosa*	[58]
13	6.70	Methyl gallate	$C_8H_8O_5$	$[M-H]^-$	183.0288	183.0293	2.73	183.0293；168.0058；140.0108；111.0078；72.9919	Organic acid	*P. lactiflora*	[52]

**14**	7.70	Dihydrophaseic acid 4-*O*-β-D-glucopyranoside	$C_{21}H_{32}O_{10}$	[M-H]$^-$	443.1911	443.1927	3.61	443.1922；250.8443；223.7933；101.0233	Organic acid	*R. glutinosa*	[32]
**15**	9.59	Oxypaeoniflorin	$C_{23}H_{28}O_{12}$	[M-H]$^-$	495.1497	495.1516	3.84	495.1509；137.0237；93.0336	Terpenoids	*P. lactiflora*	[52]
**16**	9.81	Caffeic acid*	$C_9H_8O_4$	[M-H]$^-$	179.0339	179.0344	2.79	179.0344；135.0444；90.9972；59.0128	Organic acid	*R. glutinosa*	[59]
**17**	10.38	1,6-*Bis*-*O*-(3,4,5-trihydroxybenzoyl)hexopyranose	$C_{20}H_{20}O_{14}$	[M-H]$^-$	483.0769	483.0779	2.07	483.0779；271.0461；211.0246；169.0136；125.0236	Organic acid	*P. emblica*	[60]
**18**	14.08	Paeonilactone B	$C_{10}H_{12}O_4$	[M+H]$^+$	197.0808	197.0812	2.03	197.0812；179.07055；151.0755；133.0650	Terpenoids	*P. lactiflora*	[52]
**19**	14.20	Paeoniflorin	$C_{23}H_{28}O_{11}$	[M+H]$^+$	481.1704	481.1707	0.62	319.1171；301.1066；197.0810；133.0650；105.0340	Terpenoids	*P. lactiflora*	[52]
**20**	14.66	Ethyl gallate	$C_9H_{10}O_5$	[M-H]$^-$	197.0444	197.0453	4.57	197.0451；169.0135；140.0106；125.0236；111.0079	Organic acid	*P. emblica*	[55]
**21**	14.82	Corilagin	$C_{27}H_{22}O_{18}$	[M-H]$^-$	633.0722	633.0739	2.69	633.0734；463.0524；300.9991；275.0197	Organic acid	*P. emblica*	[55]
**22**	14.96	Rehmapicroside	$C_{16}H_{26}O_8$	[M-H]$^-$	345.1544	345.1559	4.35	345.1554；301.1655；183.1017；161.0449；113.0238	Terpenoids	*R. glutinosa*	[38,61]
**23**	15.29	*Trans*-Cinnamaldehyde	C_9H_8O	[M+H]$^+$	133.0648	133.0650	1.50	133.0650；105.0703；103.0547；95.0549；79.0549	Phenylpropanoids	*R. glutinosa*	[38]
**24**	15.30	Benzoic acid	$C_7H_6O_2$	[M-H]$^-$	121.0284	121.0287	2.48	121.0287；93.0337	Organic acid	*P. lactiflora*	[52]
**25**	15.41	2-Methoxy-4-vinylphenol	$C_9H_{10}O_2$	[M+H]$^+$	151.0754	151.0756	1.32	151.0755；123.0807；105.0704；95.0860；81.0705	Phenols	*G. uralensis*	[62]
**26**	15.63	1,3,6-tri-*O*-galloylglucose	$C_{27}H_{24}O_{18}$	[M-H]$^-$	635.0879	635.0901	3.46	635.0880；313.0565；211.0254；169.0136；125.0235	Organic acid	*P. lactiflora*	[63]
**27**	15.82	Galloyloxypaeoniflorin	$C_{30}H_{32}O_{16}$	[M-H]$^-$	647.1607	647.1627	3.09	647.1618；543.9344；313.0557；169.0137；137.0237	Organic acid	*P. lactiflora*	[64]
**28**	16.17	Purpureaside C	$C_{35}H_{46}O_{20}$	[M-H]$^-$	785.2499	785.2505	0.76	785.2505；623.2203；477.1626；161.0238；133.0287	Flavonoid	*R. glutinosa*	[38]
**29**	16.20	Skimmin	$C_{15}H_{16}O_8$	[M+H]$^+$	325.0918	325.0903	-4.61	325.0903；163.0391；107.0493	Phenylpropanoids	*R. glutinosa*	[65]
**30**	16.38	Schaftoside	$C_{26}H_{28}O_{14}$	[M-H]$^-$	563.1395	563.1412	3.02	563.1407；473.1087；383.0773；353.0671；297.0769	Flavonoid	*G. uralensis*	[66]

**31**	16.71	Mudanpioside E	$C_{24}H_{30}O_{13}$	$[M-H]^-$	525.1603	525.1619	3.05	525.1604；449.1453；327.1086；121.0286	Acetophenone	*P. lactiflora*	[52]
**32**	16.77	Daidzin	$C_{21}H_{20}O_9$	$[M-H]^-$	415.1024	415.1036	2.89	415.1050；252.0429；135.0078	Flavonoid	*G. uralensis*	[14]
**33**	17.31	Jionoside A1	$C_{36}H_{48}O_{20}$	$[M-H]^-$	799.2655	799.2678	2.88	799.2671；623.2194；175.0395；160.0159	Phenylpropanoids	*R. glutinosa*	[38]
**34**	17.35	Rehmaionoside C	$C_{19}H_{32}O_8$	$[M+Na]^+$	411.1989	411.1998	2.19	411.1992；203.0527	Terpenoids	*R. glutinosa*	[38,61]
**35**	17.66	Naringeninchalcone	$C_{15}H_{12}O_5$	$[M+H]^+$	273.0758	273.0762	1.46	273.0758；153.0184；147.0442；119.0494；91.0549	Flavonoid	*G. uralensis*	[35]
**36**	17.71	Liquiritigenin*	$C_{15}H_{12}O_4$	$[M+H]^+$	257.0808	257.0811	1.17	257.0809；147.0443；137.0235；119.0496	Flavonoid	*G. uralensis*	[67]
**37**	17.75	Ellagic acid*	$C_{14}H_6O_8$	$[M-H]^-$	300.9978	300.9990	3.99	300.9990；257.0091；229.0140；185.0240	Organic acid	*P. emblica*, *P. lactiflora*	[52,37]
**38**	17.93	Liquiritigenin-7-*O*-β-D-apiofuranosyl-4′-*O*-β-D-glucopyranoside	$C_{26}H_{30}O_{13}$	$[M-H]^-$	549.1603	549.1617	2.55	549.1613；255.0662；153.0186；135.0079；119.0494	Flavonoid	*G. uralensis*	[68]
**39**	18.02	Spiraeoside	$C_{21}H_{20}O_{12}$	$[M-H]^-$	463.0871	463.0888	3.67	463.0887；301.0354；151.0029	Flavonoid	*P. lactiflora*	[52]
**40**	18.41	Quercetin-3-*O*-β-D-glucoside	$C_{21}H_{20}O_{12}$	$[M-H]^-$	463.0871	463.0888	3.67	463.0880；300.0269；271.0249；255.0296；151.0030	Flavonoid	*P. emblica*, *G. uralensis*	[37,69]
**41**	18.44	Salicylic acid	$C_7H_6O_3$	$[M-H]^-$	137.0233	137.0236	2.19	137.0236；93.0336；65.0386	Organic acid	*O. japonicus*	[70]
**42**	18.48	Galloylpaeoniflorin	$C_{30}H_{32}O_{15}$	$[M+H]^+$	633.1814	633.1824	1.58	512.9092；297.0610；197.0810；153.0184；105.0340	Terpenoids	*P. lactiflora*	[52]
**43**	19.39	Jionoside B1	$C_{37}H_{50}O_{20}$	$[M-H]^-$	813.2811	813.2835	2.95	813.2830；637.2319；175.0395；160.0159	Phenylpropanoids	*R. glutinosa*	[38]
**44**	19.90	Verbascoside	$C_{29}H_{26}O_{15}$	$[M-H]^-$	623.1970	623.1988	2.89	623.1982；461.1660；161.0237；133.0287；113.0235	Phenylpropanoids	*R. glutinosa*	[71]
**45**	19.93	Astragalin	$C_{21}H_{20}O_{11}$	$[M-H]^-$	447.0922	447.0938	3.58	447.0934；284.0328；255.0299；227.0348	Flavonoid	*G. uralensis*	[34]
**46**	19.94	Kaempferol	$C_{15}H_{10}O_6$	$[M+H]^+$	287.0550	287.0554	1.39	287.0551；213.0548；153.0183；121.0286	Flavonoid	*P. emblica*, *P. lactiflora*, *G. uralensis*	[31,72,73]

**47**	19.97	Trifolin	$C_{21}H_{20}O_{11}$	$[M+H]^+$	449.1078	449.1085	1.56	303.0498; 287.0551; 158.0519; 153.0184; 85.0290	Flavonoid	*P. emblica*	[74]
**48**	20.35	Prunin	$C_{21}H_{22}O_{10}$	$[M-H]^-$	433.1129	433.1140	2.54	271.0614; 177.0187; 151.0034; 119.0494	Flavonoid	*P. emblica*	[75]
**49**	20.40	Kaempferol-7-*O*-β-D-glucopyranoside*	$C_{21}H_{20}O_{11}$	$[M-H]^-$	447.0922	447.0938	3.58	447.0936; 285.0408; 257.0447; 169.0132; 151.0030	Flavonoid	*P. emblica*	[75]
**50**	20.45	Azelaic acid	$C_9H_{16}O_4$	$[M-H]^-$	187.0965	187.0973	4.28	187.0971; 169.0862; 143.1071; 125.0964; 97.0649	Organic acid	*O. japonicus*	[70]
**51**	21.33	Albiflorin	$C_{23}H_{28}O_{11}$	$[M+Na]^+$	503.1523	503.1528	0.99	503.1528; 381.1164; 341.0996; 219.0630; 133.0650	Terpenoids	*P. lactiflora*	[76]
**52**	22.62	Jionoside D1	$C_{30}H_{38}O_{15}$	$[M-H]^-$	637.2127	637.2147	3.14	637.2133; 461.1656; 348.7423; 175.0394; 160.0160	Phenylpropanoids	*R. glutinosa*	[38]
**53**	22.73	Ononin	$C_{22}H_{22}O_9$	$[M+H]^+$	431.1337	431.1346	2.09	431.1342; 269.0810; 237.0546; 213.0912	Flavonoid	*G. uralensis*	[67]
**54**	22.79	Formononetin	$C_{16}H_{12}O_4$	$[M+H]^+$	269.0808	269.0811	1.11	269.0810; 254.0573; 213.0909; 118.0418; 107.0493	Flavonoid	*G. uralensis*	[67]
**55**	22.96	Liquiritin*	$C_{21}H_{22}O_9$	$[M-H]^-$	417.1180	417.1197	4.08	417.1192; 255.0663; 153.0186; 135.0079; 119..0494	Flavonoid	*G. uralensis*	[67]
**56**	23.00	Aeginetic acid	$C_{15}H_{24}O_4$	$[M+H]^+$	269.1747	269.1752	1.86	269.1754; 221.1538; 159.0807	Terpenoids	*R. glutinosa*	[77]
**57**	23.04	3',6'-di-*O*-galloylpaeoniflorin	$C_{37}H_{36}O_{19}$	$[M-H]^-$	783.1767	783.1786	2.43	783.1782; 660.3563; 465.1376; 169.0136; 125.0239	Terpenoids	*P. lactiflora*	[33]
**58**	23.47	Hydrocinnamic acid	$C_9H_{10}O_2$	$[M-H]^-$	149.0597	149.0602	3.35	149.0600; 121.0285; 104.9085	Organic acid	*R. glutinosa*	[78]
**59**	23.60	N-*trans*-Feruloyltyramine	$C_{18}H_{19}NO_4$	$[M+H]^+$	314.1387	314.1392	1.59	314.1392; 177.0548; 145.0286; 121.0651	Alkaloid	*R. glutinosa*	[38]
**60**	23.61	Licochalcone B	$C_{16}H_{14}O_5$	$[M-H]^-$	285.0758	285.0771	4.56	285.0771; 270.0536; 177.0188; 150.0315	Flavonoid	*G. uralensis*	[79]
**61**	24.05	Isoliquiritigenin*	$C_{15}H_{12}O_4$	$[M+H]^+$	257.0808	257.0811	1.17	257.0808; 147.0441; 137.0234; 119.0494	Flavonoid	*G. uralensis*	[67]

**62**	25.26	Benzoyloxypaeoniflorin	$C_{30}H_{32}O_{13}$	$[M-H]^-$	599.1759	599.1777	3.00	599.1769；535.8405；281.4911；137.0236	Terpenoids	*P. lactiflora*	[33]
**63**	25.76	Quercetin*	$C_{15}H_{10}O_7$	$[M+H]^+$	303.0499	303.0503	1.32	303.0503；229.0500；153.0186；137.0229	Flavonoid	*P. emblica*	[60]
**64**	25.99	Martynoside	$C_{31}H_{40}O_{15}$	$[M-H]^-$	651.2283	651.2303	3.07	651.2299；503.7291；175.0396；160.0159	Phenylpropanoids	*R. glutinosa*	[38]
**65**	26.34	Afzelin*	$C_{21}H_{20}O_{10}$	$[M-H]^-$	431.0972	431.0986	3.25	431.0981；285.0405；257.0455；169.0135；151.0027	Flavonoid	*P. emblica*	[75]
**66**	26.50	Ophiofurospiside K	$C_{51}H_{84}O_{23}$	$[M-H]^-$	1063.5320	1063.5350	2.82	1063.5337；901.4876；699.6534	Steroid saponin	*O. japonicus*	[70]
**67**	28.73	Mudanpioside B	$C_{31}H_{34}O_{14}$	$[M-H]^-$	629.1864	629.1882	2.86	593.6018；372.5136；269.6454；121.0285	Terpenoids	*P. lactiflora*	[52]
**68**	30.67	Luteolin	$C_{15}H_{10}O_6$	$[M-H]^-$	285.0394	285.0408	4.91	285.0406	Flavonoid	*P. emblica*	[37]
**69**	31.12	Benzoylpaeoniflorin	$C_{30}H_{32}O_{12}$	$[M+H]^+$	585.1967	585.1971	0.68	353.1003；319.1172；249.0759；197.0810；105.0339	Terpenoids	*P. lactiflora*	[76]
**70**	34.46	Macedonoside A	$C_{42}H_{62}O_{17}$	$[M+H]^+$	839.4060	839.4070	1.19	469.3314；217.1589；173.1328	Triterpenoid saponins	*G. uralensis*	[80]
**71**	34.61	Licoricesaponin G2*	$C_{42}H_{62}O_{17}$	$[M-H]^-$	837.3903	837.3922	2.27	837.3915；351.0565；193.0351；113.0235	Triterpenoid saponins	*G. uralensis*	[81]
**72**	34.98	(15*Z*)-9,12,13-Trihydroxy-15-octadecenoic acid	$C_{18}H_{34}O_5$	$[M-H]^-$	329.2323	329.2334	3.34	329.2334；211.1335；171.1020；139.1120；99.0806	Triterpenoid saponins	*G. uralensis*	[67]
**73**	35.15	Parthenolide	$C_{13}H_{22}O_3$	$[M+H]^+$	249.1485	249.1485	0.00	249.1485；231.1380；213.1276；185.1330；175.0760	Terpenoids	*G. uralensis*	[67]
**74**	39.23	Glycyrrhizin	$C_{42}H_{62}O_{16}$	$[M+H]^+$	823.4110	823.4119	1.09	647.3788；471.3474；453.3365	Terpenoids	*G. uralensis*	[67]
**75**	39.26	18-β-Glycyrrhetinic acid	$C_{30}H_{46}O_4$	$[M+H]^+$	471.3469	471.3474	1.06	471.3473；317.2119；235.1688；189.1640；149.0965	Triterpenoid saponins	*G. uralensis*	[67]
**76**	39.29	Monoglucuronylglycyrrhetinic acid	$C_{36}H_{54}O_{10}$	$[M+H]^+$	647.3789	647.3797	1.24	647.3787；453.3366	Triterpenoid saponins	*G. uralensis*	[82]
**77**	40.54	Licoricesaponin H2	$C_{42}H_{62}O_{16}$	$[M-H]^-$	821.3954	821.3975	2.56	821.3959；540.8403；351.0580；193.0348；113.0233	Triterpenoid saponins	*G. uralensis*	[81]

**78**	42.65	Atractylodin	$C_{13}H_{10}O$	$[M+H]^+$	183.0804	183.0806	1.09	183.0806；113.9640；105.0339；95.0497	Polyacetylene	*G. uralensis*	[67]
**79**	43.52	Ophiopogonin D	$C_{45}H_{72}O_{18}$	$[M-H]^-$	899.4634	899.4655	2.33	853.4579；721.4168；145.0497；127.0392	Steroid saponin	*O. japonicus*	[83]
**80**	43.91	Kanzonol K	$C_{26}H_{28}O_6$	$[M+H]^+$	437.1959	437.1941	-4.12	437.1938；303.1194	Flavonoid	*G. uralensis*	[84]
**81**	44.36	Methylophiopogonanone A	$C_{19}H_{18}O_6$	$[M-H]^-$	341.1020	341.1031	3.22	341.1031；206.0580；178.0630	Flavonoid	*O. japonicus*	[70]
**82**	49.27	Oleamide	$C_{18}H_{35}NO$	$[M+H]^+$	282.2791	282.2794	1.06	282.2792；265.2526；247.2420；135.1174；95.0859	Fatty acid amides	*G. uralensis*	[67]
**83**	49.38	Hexadecanamide	$C_{16}H_{33}NO$	$[M+H]^+$	256.2635	256.2637	0.78	256.2640；102.0917；88.0762；57.0707	Fatty acid amides	*G. uralensis*	[67]
**84**	56.08	Erucamide	$C_{22}H_{43}NO$	$[M+H]^+$	338.3417	338.3420	0.89	338.3416；321.3150；303.3050；97.1017；69.0706	Fatty acid amides	*G. uralensis*	[67]

Note: * indicates compounds confirmed by comparison with reference standards.

Table S2. Molecular docking binding energies and ligand efficiency of serum-absorbed prototype constituents against STAT3 and EGFR.

Compound	Heavy Atoms	STAT3		EGFR	
		Binding energy (kcal/mol ± SD)*	Ligand efficiency	Binding energy (kcal/mol ± SD)*	Ligand efficiency
**7**	15	-4.84 ± 0.17	0.32	-5.12 ± 0.16	0.34
**9**	9	-4.90 ± 0.17	0.54	-4.60 ± 0.07	0.51
**22**	24	-6.74 ± 0.27	0.28	-6.66 ± 0.13	0.28
**36**	19	-6.78 ± 0.27	0.36	-6.64 ± 0.09	0.35
**37**	22	-8.10 ± 0.27	0.37	-8.06 ± 0.19	0.37
**38**	39	-7.80 ± 0.26	0.20	-8.02 ± 0.16	0.21
**41**	10	-5.26 ± 0.22	0.53	-4.96 ± 0.05	0.50
**49**	32	-7.62 ± 0.22	0.24	-7.96 ± 0.29	0.25
**55**	30	-7.36 ± 0.15	0.25	-7.54 ± 0.11	0.25
**72**	23	-5.14 ± 0.15	0.22	-5.16 ± 0.13	0.22
**80**	32	-7.06 ± 0.15	0.22	-7.64 ± 0.21	0.24
**82**	20	-4.04 ± 0.19	0.20	-4.50 ± 0.10	0.23
**83**	18	-3.98 ± 0.16	0.22	-4.14 ± 0.05	0.23
**84**	24	-4.16 ± 0.27	0.17	-4.28 ± 0.08	0.18

*Mean ± SD of five independent runs.

Table S3. Predicted drug-likeness and physicochemical properties of the serum-absorbed prototype constituents.

Compound	Formula	Molecular Weight	Rotatable bonds	H-bond I-acceptors	H-bond I-donors	iLOGP	XLOGP3	WLOGP	Silicos-IT Log P	Consensus Log P	GI absorption	Lipinski violations
**7**	$C_9H_{17}NO_5$	219.23	7	5	4	0.95	-1.08	-1.04	-0.44	-0.48	High	0
**9**	$C_6H_6O_3$	126.11	0	3	3	0.97	0.52	0.8	0.43	0.58	High	0
**22**	$C_{16}H_{26}O_8$	346.37	4	8	5	1.98	-0.58	-0.61	-0.55	-0.12	Low	0
**36**	$C_{15}H_{12}O_4$	256.25	1	4	2	1.67	2.3	2.48	2.55	2.05	High	0
**37**	$C_{14}H_6O_8$	302.19	0	8	4	0.79	1.1	1.31	1.67	1	High	0
**38**	$C_{26}H_{30}O_{13}$	550.51	7	13	7	2.36	-1.04	-1.93	-0.72	-0.77	Low	3
**41**	$C_7H_6O_3$	138.12	1	3	2	1.13	2.26	1.09	0.74	1.24	High	0
**49**	$C_{21}H_{20}O_{11}$	448.38	4	11	7	1.36	0.72	-0.24	-0.12	-0.08	Low	2
**55**	$C_{21}H_{22}O_9$	418.39	4	9	5	1.46	0.39	-0.05	0.36	0.25	Low	0
**72**	$C_{18}H_{34}O_5$	330.46	15	5	4	3.15	3.05	3.02	3.75	3	High	0
**80**	$C_{26}H_{28}O_6$	436.5	6	6	3	4.33	6.49	5.6	6.1	4.96	High	0
**82**	$C_{18}H_{35}NO$	281.48	15	1	1	4.1	6.99	5.51	5.71	5.29	High	1
**83**	$C_{16}H_{33}NO$	255.44	14	1	1	3.87	6.52	4.95	5.01	4.83	High	0
**84**	$C_{22}H_{43}NO$	337.58	19	1	1	5.02	9.16	7.07	7.47	6.76	Low	1

Table S4. In silico ADMET profiles of the serum-absorbed prototype constituents.

Compound	Caco-2	HIA	Pgp inhibition	PPB	Pure water solubility mg/L
**7**	0.22	36.54	Non	7.14	280359.00
**9**	20.71	77.41	Non	100.00	52446.30
**22**	13.53	31.52	Non	45.21	12623.20
**36**	17.65	92.36	Non	97.99	164.19
**37**	20.52	56.31	Non	66.53	1995.06
**38**	9.28	22.60	Inhibitor	41.14	53.17
**41**	20.31	88.14	Non	8.77	13576.70
**49**	5.64	25.17	Non	60.77	90.01
**55**	8.49	65.80	Non	71.69	121.01
**72**	5.92	86.48	Inhibitor	100.00	399.78
**80**	14.89	92.74	Inhibitor	98.37	0.47
**82**	29.31	95.33	Inhibitor	100.00	0.66
**83**	26.39	95.13	Inhibitor	100.00	1.22
**84**	35.78	95.57	Inhibitor	100.00	0.02
